# The global trends and regional differences in incidence and mortality of hepatitis A from 1990 to 2019 and implications for its prevention

**DOI:** 10.1007/s12072-021-10232-4

**Published:** 2021-08-03

**Authors:** Guiying Cao, Wenzhan Jing, Jue Liu, Min Liu

**Affiliations:** Department of Epidemiology and Biostatistics, School of Public Health, Peking University, Haidian District, No. 38 Xueyuan Road, Beijing, 100191 China

**Keywords:** Hepatitis A, Age-standardized incidence rate, Age-standardized mortality rate, Trend, Global burden of disease, Prevention

## Abstract

**Background and purpose:**

Despite decades of improved sanitation and hygiene measures and vaccine introduction, hepatitis A has been spread through numerous outbreaks globally. We used data from the Global Burden of Disease (GBD) study to quantify hepatitis A burden at the global, regional and national levels.

**Methods:**

Annual incident cases, deaths, age-standardized incidence rates (ASIRs), and age-standardized mortality rates (ASMRs) of hepatitis A between 1990 and 2019 were derived from the GBD study 2019. Percentage changes of cases and deaths, and estimated annual percentage changes (EAPCs) of ASIRs and ASMRs were calculated to quantify their temporal trends.

**Results:**

Global hepatitis A incident cases increased by 13.90% from 139.54 million in 1990 to 158.94 million in 2019. ASIR of hepatitis A remained stable (EAPC = 0.00, 95% CI −0.01 to 0.01), whereas ASMR decreased (EAPC = −4.63, 95% CI −4.94 to −4.32) between 1990 and 2019. ASIR increased in low (EAPC = 0.09, 95% CI 0.04 to 0.14) and low-middle (EAPC = 0.04, 95% CI 0.03 to 0.06) socio-demographic index (SDI) regions. For GBD regions, the most significant increases of ASIR were detected in high-income Asia Pacific (EAPC = 0.53, 95% CI 0.41 to 0.66), Oceania (EAPC = 0.31, 95% CI 0.25 to 0.36), and Australasia (EAPC = 0.28, 95% CI 0.13 to 0.44). EAPC of ASIR was positively associated with SDI value in countries and territories with SDI value ≥ 0.7 (*ρ* = −0.310, *p* < 0.001).

**Conclusion:**

There is an unfavorable trend that hepatitis A is still pending in hyperendemic regions and is emerging in low endemic regions. These highlight the need of targeted and specific strategies to eliminate hepatitis A, such as sanitation measures and a comprehensive plan for surveillance and vaccination against hepatitis A.

**Supplementary Information:**

The online version contains supplementary material available at 10.1007/s12072-021-10232-4.

## Introduction

Hepatitis A caused by hepatitis A virus (HAV) is the most common form of acute viral hepatitis globally [1]. HAV first identified by Feinstone and colleagues, is a member of the *Hepatovirus* genus of the family *Picornaviridae* [2, 3]. HAV is transmitted primarily through ingestion of food and water contaminated with the feces of an infected person or through close physical contact with an infectious person [4]. The World Health Organization (WHO) estimates more than 100 million HAV infections, causing approximately 1.5 million clinical cases of hepatitis A each year [5]. Symptomatic patients of hepatitis A can experience mild to severe illness, including nausea, vomiting, malaise, diarrhea, abdominal discomfort, fever, dark-colored urine, jaundice, and fulminant hepatitis, which is often fatal [6, 7]. HAV infection is the second largest cause of fulminant viral hepatitis characterized by massive hepatocyte necrosis and inflammatory infiltrate after hepatitis B virus (HBV) infection [8]. In addition, superinfection of HAV with HBV is also a health problem in regions with high endemicity levels both of HAV and HBV [9].

After transmission of HAV, the virus enters the blood stream to gain access to the liver, the primary site of its replication, and induces liver injury. The pathogenesis of hepatitis A and the mechanisms underlying hepatocellular injury remain incompletely understood. Recent evidence suggests that gangliosides function as essential endosomal receptors for quasi-enveloped and naked HAV [10]. In addition, one previous study has reported that free fatty acids or high-concentration glucose enhances HAV replication in association with a reduction in glucose-regulated protein 78 expression [11]. Virus-specific cytotoxic T lymphocytes play a pivotal role in clearing virus-infected cells but also a major driver for HAV-associated immunopathology that may also cause liver injury [11]. Natural killer (NK) cells, NKT cells, and non-HAV-specific CD8^+^ T cells have been reported to contribute to liver injury [11–13]. In addition, intrinsic apoptosis of virus-infected hepatocytes has been implicated as a cause of liver injury in a murine model of HAV infection [14]. Finally, the severity of HAV-induced liver injury may be linked to genetic variations in both host and viral factors [11, 15]. Since hepatitis A is clinically indistinguishable from diseases caused by other hepatitis viruses, specific serum markers and nucleic acid detection techniques are required to make the diagnosis [16]. Immunoglobulin M anti-HAV increases in the acute setting and confirms recent exposure to HAV, whereas immunoglobulin G anti-HAV is elevated during convalescence and indicates past exposure. HAV nucleic acid sequencing is performed on polymerase chain reaction products to provide the ultimate means of identifying and characterizing the organism.

Hepatitis A vaccine is highly immunogenic and well tolerated in preventing hepatitis A [17]. Several randomized controlled trials reported that the efficacy of hepatitis A vaccine used for pre-exposure prophylaxis and post-exposure prophylaxis ranged from 95 to 100% among children [18–21]. However, hepatitis A vaccine was used or planned to introduce in routine immunization of children in only 34 countries as of May 2019 worldwide. In addition to the introduction of hepatitis A vaccine, decades of improved sanitation and hygiene measures contribute to combat hepatitis A. The sanitary conditions and hygienic practices were good in high-income countries but still poor in low- and middle-income countries. However, hepatitis A outbreaks frequently occurred in countries regardless of their income in recent years [22–24]. In high-income countries, imported frozen produce items and infected travelers from endemic regions are associated with hepatitis A outbreaks [24–26]. In addition, hepatitis A outbreaks frequently occurred among the men who have sex with men (MSM) and persons who injected drugs (PWIDs) in high-income countries [27, 28]. Frequent international trades and travels in middle-income countries play important roles in outbreaks of hepatitis A and global transmissions of HAV [24]. For low-income countries, the endemicity levels of hepatitis A are still high due to poor sanitary conditions and hygienic practices [24]. The prevention of hepatitis A is facing more and more complex situations, especially with globalization processes. Therefore, understanding the endemicity levels of hepatitis A at the global, regional and national levels and targeted strategies for its prevention were essential for achieving the goal of eliminating viral hepatitis by 2030.

In this current study, we extracted detailed data of the incidence and mortality of hepatitis A from the Global Burden of Disease (GBD) Study 2019 to assess the temporal trends in incidence and mortality of hepatitis A at global, regional, and national levels from 1990 to 2019. Our study can extend and complement the previous study [29], while also providing a more comprehensive perspective in the design of targeted strategies in hepatitis A prevention tailored to different countries.

## Methods

### Data source

This study used data of annual incident cases, deaths, age-standardized incidence rates (ASIRs), and age-standardized mortality rates (ASMRs) of hepatitis A from 1990 to 2019, by sex, age, and location, collected from the Global Health Data Exchange (GHDx) query tool (http://ghdx.healthdata.org/gbd-results-tool) [30]. Data were available from a total of 204 countries and territories, and these were categorized into 5 regions in terms of socio-demographic index (SDI) and 21 GBD regions according to geographical contiguity. The 21 GBD regions, including 5 low-income regions, 12 middle-income regions, and 4 high-income regions, are listed in Table S1. Specific methods of GBD study 2019 estimation process for the incidence of hepatitis A were described elsewhere [31]. Briefly, age-specific anti-HAV immunoglobulin G seroprevalence data from population-based studies and surveys were reviewed to estimate the incidence of hepatitis A using the Bayesian meta-regression tool by DisMod-MR 2.1 model [31]. The number of populations in 204 countries and territories of GBD study was collected from the United Nations, Department of Economic and Social Affairs, Population Division (2019) (https://population.un.org/wpp/) [24].

### SDI

The SDI is a composite indicator of development status strongly correlated with health outcomes [30]. It is the geometric mean of 0 to 1 indices of lag distributed income per capita, average years of schooling for those ages 15 and older, and total fertility rate under the age of 25. A location with an SDI of 0 indicates a theoretical minimum level of development status relevant to health outcomes, while a location with an SDI of 1 indicates a theoretical maximum level [30]. According to SDI values in 2019, the 204 countries and territories were divided into five regions, including low, low-middle, middle, high-middle, and high SDI regions. The SDI values of 204 countries and territories in 2019 were shown in Table S2.

### Statistical analysis

The percentage changes in hepatitis A incident cases and deaths, and the estimated annual percentage changes (EAPCs) of ASIRs and ASMRs were calculated to quantify the trends in incidence and mortality of hepatitis A. To compare the incidence and mortality rates of hepatitis A across different populations, the ASIRs and ASMRs were carried out by applying the age-specific rates for each location, sex and year to a GBD World Standard Population to adjust for potential confounding of age structure [32]. The percentage changes in hepatitis A incident cases from 1990 to 2019 were calculated by the equation: Percentage changes=$\frac{\mathrm{Incident cases in }2019 -\mathrm{ Incident cases in }1990}{\mathrm{Incident cases in }1990}\times$ 100%. Similar with the calculation of percentage changes in incident cases, the percentage changes in hepatitis A death and number of populations were calculated. EAPC is a summary and widely used measure of the ASIR and ASMR tend over a specified time interval. A regression line was fitted to the natural logarithm of the ASIR, i.e., *y* = α + β*x* + *ε*, where *y* = ln (ASIR) and *x* = calendar year. EAPC was calculated as $100\times {(e}^{\beta }-1)$ and its 95% confidence interval (CI) was calculated to reflect the temporal trend in ASIR. Similar with the calculation of EAPC of ASIR, the EAPC of ASMR was calculated. The trends in ASIR and ASMR were reflected in the EAPC value and its 95% CI: ASIR and ASMR are in an upward trend when the EAPC and the lower boundary of the 95% CI are positive; conversely, ASIR and ASMR are in a downward trend when EAPC and the upper boundary of the 95% CI are negative.

Moreover, the correlations between EAPC and ASIR (1990) as well as SDI values (2019) in different countries and territories were evaluated by Pearson correlation analyses to define the potential factors affecting EAPC. The polynomial curves were also modeled. All analyses were conducted with SAS 9.4 (SAS Institute, Inc., Cary, NC) and Origin 2019b. A two-tailed *p* value less than 0.05 was considered statistically significant.

## Results

### Global trends in incidence and mortality rates of hepatitis A

Globally, the number of hepatitis A incident cases increased by 13.90% from 139.54 million in 1990 to 158.94 million in 2019, whereas the number of hepatitis A deaths decreased by 63.61% from 10.79 in 1990 thousand to 3.93 thousand in 2019 (Table 1). The overall ASIR of hepatitis A remained stable (EAPC = 0.00, 95% CI −0.01 to 0.01) from 2263.97 per 100,000 in 1990 to 2272.08 per 100,000 in 2019, whereas the overall ASMR of hepatitis A decreased (EAPC = −4.63, 95% CI −4.94 to −4.32) from 2.07 per 100,000 in 1990 to 0.52 per 100,000 in 2019 (Table 2).

**Table 1:** The incident cases and deaths of hepatitis A and population in 1990 and 2019 and their percentage change from 1990 to 2019

Characteristics	Incident cases			Deaths			Population		
	1990 No. × 10^5^ (95% UI)	2019 No. × 10^5^ (95% UI)	Percentage change (%)	1990 No. × 10^3^ (95% UI)	2019 No. × 10^3^ (95% UI)	Percentage change (%)	1990 No. × 10^5^	2019 No. × 10^5^	Percentage change (%)
Overall	1395.44 (1303.39–1484.48)	1589.44 (1492.31–1690.85)	13.90	107.92 (82.49–133.25)	39.28 (27.99–52.35)	− 63.61	53182.09	77010.07	44.80
SDI									
Low	223.05 (208.56–237.30)	390.12 (364.06–415.51)	74.90	22.58 (14.57–31.50)	13.16 (9.03–17.94)	− 41.73	4272.77	9416.38	120.38
Low-middle	377.05 (352.73–402.77)	421.56 (395.19–449.50)	11.80	52.20 (39.62–64.40)	19.79 (13.43–27.76)	− 62.08	13911.82	22561.46	62.17
Middle	469.65 (436.39–502.51)	434.57 (404.34–464.46)	− 7.47	27.50 (20.60–34.37)	5.40 (3.07–8.78)	− 80.37	21411.40	28885.04	34.90
Middle-high	235.45 (217.05–253.19)	200.58 (185.08–215.46)	− 14.81	5.29 (3.94–6.62)	0.80 (0.47–1.47)	− 84.84	6120.66	7067.99	15.48
High	89.47 (81.33–97.53)	95.44 (87.17–103.48)	6.67	0.33 (0.26–0.44)	0.11 (0.08–0.15)	− 65.65	7465.43	9079.20	21.62
GBD region									
High-income Asia Pacific	17.76 (16.09–19.35)	15.63 (14.23–17.09)	− 12.03	0.05 (0.04–0.06)	0.01 (0.01–0.02)	− 68.57	1706.95	1843.23	7.98
Central Asia	22.42 (20.65–24.08)	23.51 (21.70–25.27)	4.87	0.82 (0.61–1.04)	0.08 (0.05–0.10)	− 90.54	684.98	934.39	36.41
East Asia	287.42 (265.07–309.31)	200.07 (182.44–216.67)	− 30.39	12.90 (7.97–17.54)	0.63 (0.43–0.87)	− 95.12	12176.55	14832.24	21.81
South Asia	360.26 (335.30–388.00)	420.60 (388.3–453.03)	16.75	67.09 (50.34–81.66)	27.27 (17.74–39.71)	− 59.36	11035.34	17754.01	60.88
Southeast Asia	120.29 (109.37–130.96)	121.81 (111.18–132.18)	1.26	8.64 (4.74–16.21)	2.29 (0.87–4.21)	− 73.53	4598.68	6789.96	47.65
Australasia	2.02 (1.80–2.26)	2.81 (2.49–3.10)	38.64	0.00 (0.00–0.00)	0.00 (0.00–0.00)	64.55	203.59	299.86	47.29
Caribbean	9.78 (9.01–10.55)	10.16 (9.33–10.94)	3.91	0.17 (0.10–0.24)	0.06 (0.03–0.09)	− 63.85	342.60	437.63	27.74
Central Europe	19.80 (17.82–21.57)	12.93 (11.75–14.06)	− 34.70	0.09 (0.05–0.12)	0.00 (0.00–0.01)	− 95.50	1229.58	1139.58	− 7.32
Eastern Europe	37.79 (34.26–41.00)	29.15 (26.54–31.49)	− 22.88	0.04 (0.03–0.08)	0.01 (0.00–0.01)	− 77.66	2214.37	2093.54	− 5.46
Western Europe	35.82 (32.97–38.77)	37.00 (33.75–40.31)	3.28	0.11 (0.08–0.17)	0.07 (0.04–0.09)	− 35.12	3813.61	4332.11	13.60
Andean Latin America	12.61 (11.71–13.54)	15.76 (14.61–16.94)	24.97	0.17 (0.12–0.22)	0.03 (0.02–0.04)	− 81.29	391.67	613.97	56.76
Central Latin America	55.94 (52.08–59.83)	57.35 (53.68–61.06)	2.52	0.31 (0.27–0.37)	0.15 (0.12–0.19)	− 52.60	1659.31	2560.51	54.31
Southern Latin America	12.52 (11.27–13.61)	11.84 (10.63–12.99)	− 5.44	0.09 (0.07–0.10)	0.01 (0.01–0.02)	− 84.42	490.03	671.94	37.12
Tropical Latin America	39.36 (36.37–42.56)	38.30 (34.98–41.31)	− 2.68	0.21 (0.17–0.30)	0.06 (0.04–0.09)	− 72.55	1532.27	2180.94	42.33
North Africa and Middle East	117.34 (108.34–126.35)	145.52 (134.95–155.99)	24.02	7.57 (2.59–12.69)	2.18 (0.78–4.37)	− 71.21	3352.15	6107.59	82.20
High-income North America	28.91 (25.85–31.96)	34.87 (31.43–38.22)	20.63	0.09 (0.08–0.13)	0.05 (0.04–0.07)	− 42.92	2797.17	3665.33	31.04
Oceania	1.88 (1.69–2.08)	3.91 (3.51–4.29)	107.85	0.08 (0.02–0.15)	0.06 (0.03–0.12)	− 24.72	65.56	115.68	76.46
Central Sub-Saharan Africa	24.68 (22.71–26.55)	49.09 (45.35–52.63)	98.94	1.36 (0.10–3.26)	0.71 (0.15–1.47)	− 48.05	529.93	1322.70	149.60
Eastern Sub-Saharan Africa	87.90 (82.02–93.58)	163.24 (151.74–173.83)	85.71	4.45 (2.42–6.29)	3.96 (2.06–6.79)	− 10.92	1849.04	4167.37	125.38
Southern Sub-Saharan Africa	17.91 (16.61–19.16)	20.74 (19.12–22.26)	15.85	0.19 (0.05–0.40)	0.08 (0.05–0.13)	− 56.03	524.79	812.75	54.87
Western Sub-Saharan Africa	83.03 (77.09–88.91)	175.15 (162.47–187.36)	110.95	3.49 (1.19–7.16)	1.56 (0.88–2.98)	− 55.38	1983.91	4334.72	118.49

*GBD* Global Burden of Disease, *SDI* Socio-Demographic Index, *UI* uncertainty interval

**Table 2:** The ASIRs and ASMRs of hepatitis A in 1990 and 2019 and their temporal trends from 1990 to 2019

Characteristics	ASIR per 100,000			ASMR per 100,000		
	1990 No. (95% UI)	2019 No. (95% UI)	EAPC No. (95% CI)	1990 No. (95% UI)	2019 No. (95% UI)	EAPC No. (95% CI)
Overall	2263.97 (2120.55, 2404.67)	2272.08 (2121.79, 2421.80)	0.00 (− 0.01, 0.01)	2.07 (1.55, 2.56)	0.52 (0.37, 0.69)	− 4.63 (− 4.94, − 4.32)
SDI						
Low	2435.07 (2274.58, 2597.09)	2379.07 (2215.87, 2535.86)	0.09 (0.04, 0.14)	5.31 (3.09, 7.71)	1.62 (1.07, 2.19)	− 4.32 (− 4.74, − 3.91)
Low-middle	2326.27 (2178.11, 2490.21)	2339.91 (2185.20, 2499.61)	0.04 (0.03, 0.06)	5.10 (3.86, 6.42)	1.25 (0.86, 1.72)	− 4.79 (− 5.04, − 4.54)
Middle	2302.94 (2143.00, 2461.55)	2137.82 (1972.97, 2300.65)	− 0.10 (− 0.15, − 0.05)	1.83 (1.32, 2.34)	0.24 (0.13, 0.38)	− 6.90 (− 6.96, − 6.84)
Middle-high	2132.59 (1958.05, 2303.65)	1963.64 (1777.60, 2144.44)	− 0.35 (− 0.38, − 0.31)	0.49 (0.36, 0.61)	0.06 (0.03, 0.10)	− 7.56 (− 7.76, − 7.37)
High	1248.33 (1115.17, 1375.23)	1238.15 (1104.84, 1372.08)	0.02 (− 0.03, 0.06)	0.04 (0.03, 0.05)	0.01 (0.01, 0.01)	− 6.21 (− 6.63, − 5.80)
GBD region						
High-income Asia Pacific	1199.51 (1067.68, 1331.80)	1253.14 (1096.36, 1415.93)	0.53 (0.41, 0.66)	0.03 (0.02, 0.04)	0.01 (0.00, 0.01)	− 6.22 (− 6.62, − 5.82)
Central Asia	2547.44 (2364.30, 2720.15)	2458.57 (2270.29, 2642.65)	− 0.17 (− 0.20, − 0.15)	1.01 (0.77, 1.26)	0.09 (0.05, 0.12)	− 8.22 (− 8.37, − 8.08)
East Asia	2288.85 (2101.12, 2473.66)	1955.43 (1752.31, 2152.42)	− 0.47 (− 0.53, − 0.42)	1.25 (0.78, 1.70)	0.03 (0.02, 0.05)	− 12.76 (− 13.47, − 12.04)
South Asia	2262.15 (2091.44, 2450.07)	2396.09 (2221.00, 2575.73)	0.19 (0.17, 0.21)	6.53 (4.99, 8.18)	1.67 (1.10, 2.44)	− 4.60 (− 4.93, − 4.26)
Southeast Asia	2064.02 (1883.19, 2243.76)	1980.23 (1791.74, 2166.75)	− 0.11 (− 0.16, − 0.06)	2.46 (1.23, 4.77)	0.36 (0.14, 0.66)	− 6.54 (− 6.71, − 6.37)
Australasia	1081.36 (953.42, 1219.98)	1172.06 (1014.89, 1319.31)	0.28 (0.13, 0.44)	0.01 (0.00, 0.01)	0.01 (0.00, 0.01)	− 1.59 (− 2.56, − 0.60)
Caribbean	2416.16 (2233.05, 2598.65)	2393.01 (2181.45, 2592.20)	− 0.06 (− 0.08, − 0.04)	0.47 (0.30, 0.66)	0.13 (0.05, 0.20)	− 4.58 (− 5.03, − 4.13)
Central Europe	1888.21 (1686.40, 2079.67)	1724.72 (1534.73, 1911.62)	− 0.22 (− 0.29, − 0.15)	0.08 (0.05, 0.12)	0.00 (0.00, 0.00)	− 10.88 (− 11.80, − 9.94)
Eastern Europe	1984.65 (1780.53, 2168.39)	2012.35 (1812.10, 2195.27)	− 0.23 (− 0.35, − 0.11)	0.02 (0.01, 0.04)	0.00 (0.00, 0.01)	− 6.34 (− 6.91, − 5.76)
Western Europe	1090.57 (982.54, 1201.07)	1083.39 (961.16, 1209.27)	0.01 (− 0.01, 0.04)	0.02 (0.01, 0.03)	0.01 (0.00, 0.01)	− 2.45 (− 3.09, − 1.81)
Andean Latin America	2424.94 (2258.10, 2595.93)	2448.14 (2266.14, 2633.15)	0.03 (0.01, 0.05)	0.53 (0.38, 0.68)	0.05 (0.04, 0.07)	− 8.10 (− 8.77, − 7.43)
Central Latin America	2510.55 (2337.69, 2687.12)	2476.32 (2306.59, 2650.84)	− 0.02 (− 0.12, 0.07)	0.20 (0.18, 0.24)	0.06 (0.05, 0.08)	− 3.61 (− 3.87, − 3.36)
Southern Latin America	2433.41 (2190.42, 2643.11)	2084.72 (1847.54, 2315.62)	− 0.61 (− 0.65, − 0.56)	0.19 (0.15, 0.22)	0.02 (0.01, 0.03)	− 8.65 (− 9.32, − 7.98)
Tropical Latin America	2177.36 (2006.98, 2354.96)	2004.49 (1806.14, 2194.00)	− 0.63 (− 0.75, − 0.51)	0.16 (0.12, 0.22)	0.03 (0.02, 0.04)	− 6.44 (− 6.69, − 6.20)
North Africa and Middle East	2346.16 (2175.08, 2520.42)	2341.72 (2165.93, 2517.09)	− 0.06 (− 0.09, − 0.04)	2.78 (0.94, 4.48)	0.42 (0.15, 0.86)	− 6.97 (− 7.41, − 6.53)
High-income North America	1134.75 (1003.57, 1265.13)	1180.02 (1042.22, 1317.73)	− 0.02 (− 0.13, 0.09)	0.03 (0.02, 0.04)	0.01 (0.01, 0.01)	− 5.56 (− 6.42, − 4.69)
Oceania	2124.87 (1928.54, 2323.02)	2312.83 (2096.23, 2514.24)	0.31 (0.25, 0.36)	1.62 (0.45, 3.05)	0.56 (0.24, 1.16)	− 3.28 (− 3.44, − 3.13)
Central Sub-Saharan Africa	2587.18 (2417.58, 2752.66)	2543.07 (2368.10, 2713.33)	− 0.04 (− 0.05, − 0.03)	3.88 (0.26, 8.95)	0.94 (0.19, 1.82)	− 4.98 (− 5.87, − 4.09)
Eastern Sub-Saharan Africa	2514.24 (2338.12, 2700.57)	2625.34 (2425.49, 2802.26)	0.10 (0.08, 0.12)	4.09 (2.27, 6.16)	1.91 (0.93, 3.16)	− 2.70 (− 2.97, − 2.42)
Southern Sub-Saharan Africa	2511.09 (2320.54, 2701.95)	2526.16 (2333.32, 2706.97)	0.01 (0.00, 0.02)	0.44 (0.11, 0.86)	0.13 (0.08, 0.20)	− 2.86 (− 3.22, − 2.50)
Western Sub-Saharan Africa	2546.44 (2371.18, 2713.40)	2552.05 (2379.50, 2723.25)	0.00 (− 0.01, 0.01)	2.74 (0.91, 5.59)	0.54 (0.31, 1.04)	− 6.13 (− 6.53 to − 5.72)

*ASIR* age-standardized incidence rate, *ASMR* age-standardized mortality rate, *CI* confidence interval, *EAPC* estimated annual percentage change, *GBD* Global Burden of Disease, *SDI* Socio-Demographic Index, *UI* uncertainty interval

**Fig. 1:**
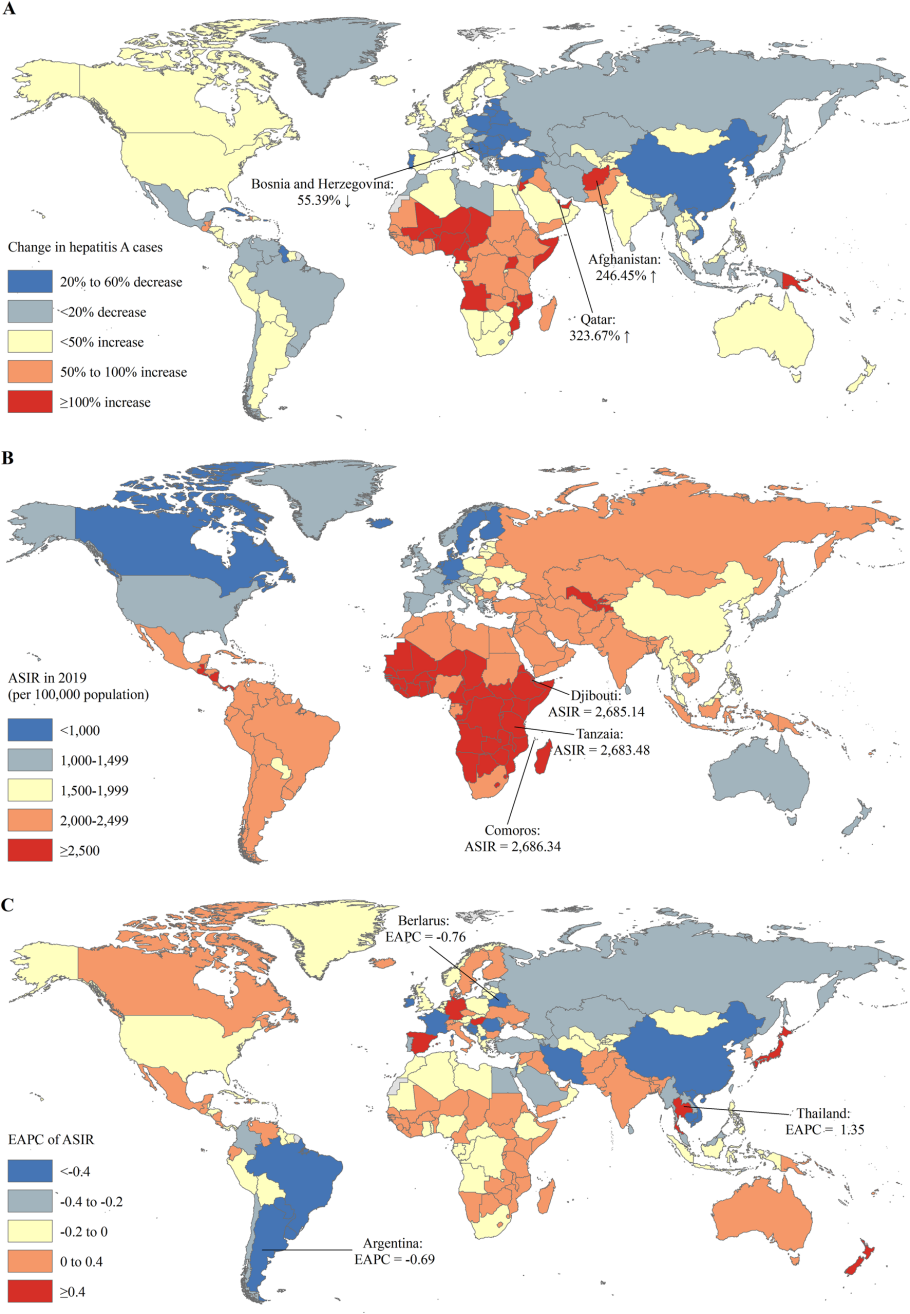
The global trends in incidence of hepatitis A in 204 countries and territories. **A** The ASIRs of hepatitis A in 2019; **B** the relative change in incident cases of hepatitis A between 1990 and 2019; **C** the EAPCs of hepatitis A ASIRs from 1990 to 2019. *ASIR* age-standardized incidence rate, *EAPC* estimated annual percentage change

The absolute number of hepatitis A incident cases in India (30.39 million) and China (19.37 million) approximately accounted for one-third of hepatitis A incident cases of the global (158.94 million) in 2019 (Table S2). The countries with the most pronounced increase of hepatitis A incident cases were Qatar (323.67%) and Afghanistan (246.45%) (Table S2 and Fig. 1A). The ASIR of hepatitis A varies considerably across the world, with the highest ASIR in Comoros (2686.34 per 100,000 in 2019), followed by Djibouti and Tanzania (Fig. 1B). The ASIRs were deemed in an increasing trend in 65 countries or territories, with the largest increase in Thailand (EAPC = 1.35; 95% CI 0.99 to 1.71), followed by Japan and Germany (Fig. 1C and Table S2). The ASRs remained stable in 35 countries or territories, including the United States of America (USA), Mexico, and Singapore. The remaining 65 countries or territories showed a decreasing trend in ASRs, with the highest decrease in Belarus (EAPC = −0.76; 95% CI −0.89 to −0.64), followed by Argentina and Romania (Fig. 1C and Table S2).

### Trends in incidence and mortality rates of hepatitis A across five SDI regions

**Fig. 2:**
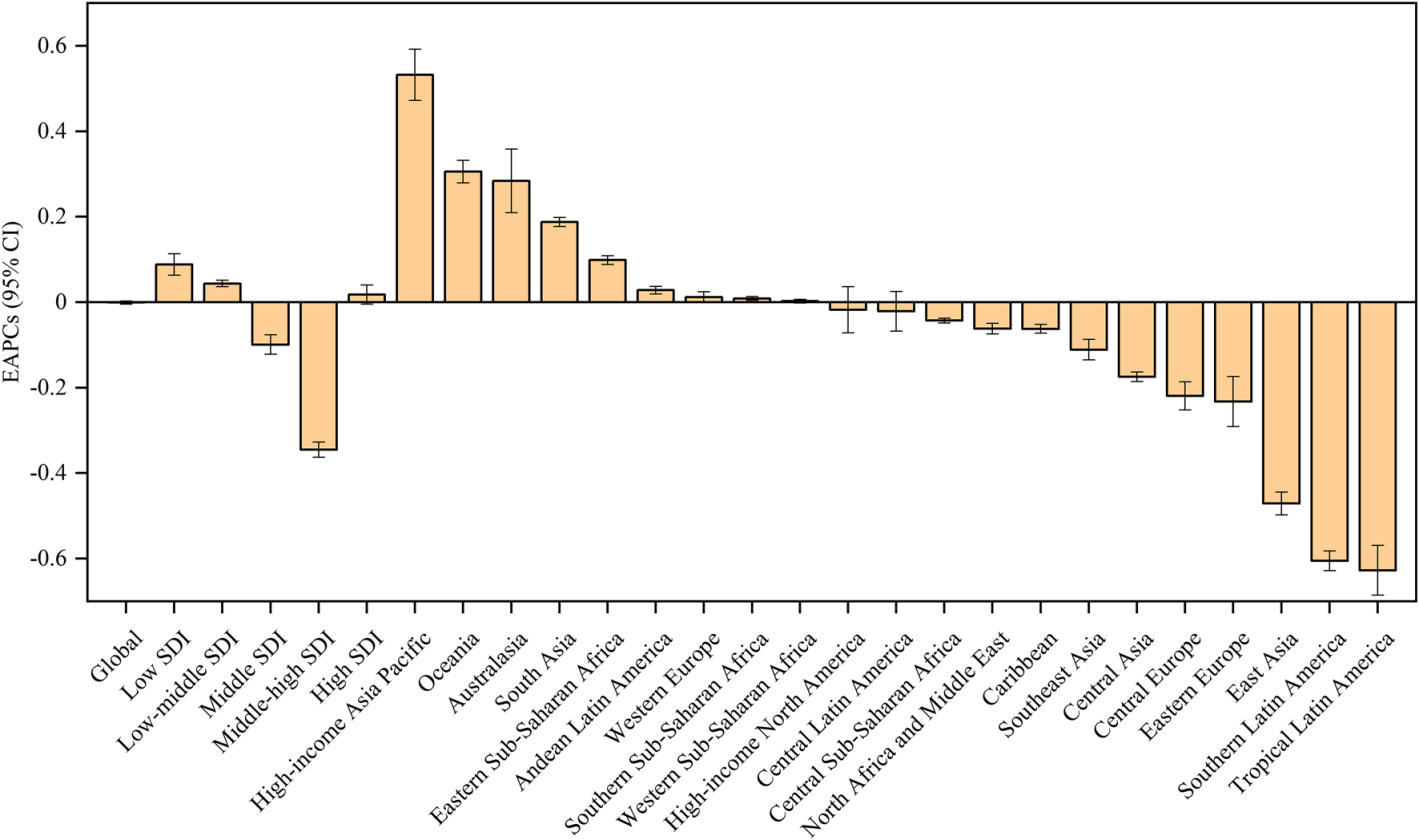
The EAPCs of hepatitis A ASIRs from 1990 to 2017 at regional level. *ASIR* age-standardized incidence rate, *EAPC* estimated annual percentage change, *SDI* socio-demographic index

For the five SDI regions, the number of hepatitis A incident cases increased in low (74.90%), low-middle (11.80%), and high (6.67%) SDI regions but decreased in middle (7.74%) and middle-high (14.81%) SDI regions (Table 1). The number of hepatitis A deaths decreased in all five SDI regions, with the largest decreased in middle-high SDI region (84.84%) (Table 1). The ASIR increased in low (EAPC = 0.09; 95% CI 0.04 to 0.14) and low-middle (EAPC = 0.04; 95% CI 0.03 to 0.06) SDI regions but decreased in middle (EAPC = −0.10; 95% CI −0.15 to −0.05) and middle-high (EAPC = -0.35; 95% CI: −0.38 to −0.31) SDI region (Table 2). The ASIR remained stable in high SDI regions (EAPC = 0.02; 95% CI −0.03 to 0.06). The ASMR decreased in all 5 SDI regions, with the largest decrease in middle-high SDI region (EAPC = −7.56; 95% CI −7.76 to −7.37) (Fig. 2).

**Fig. 3:**
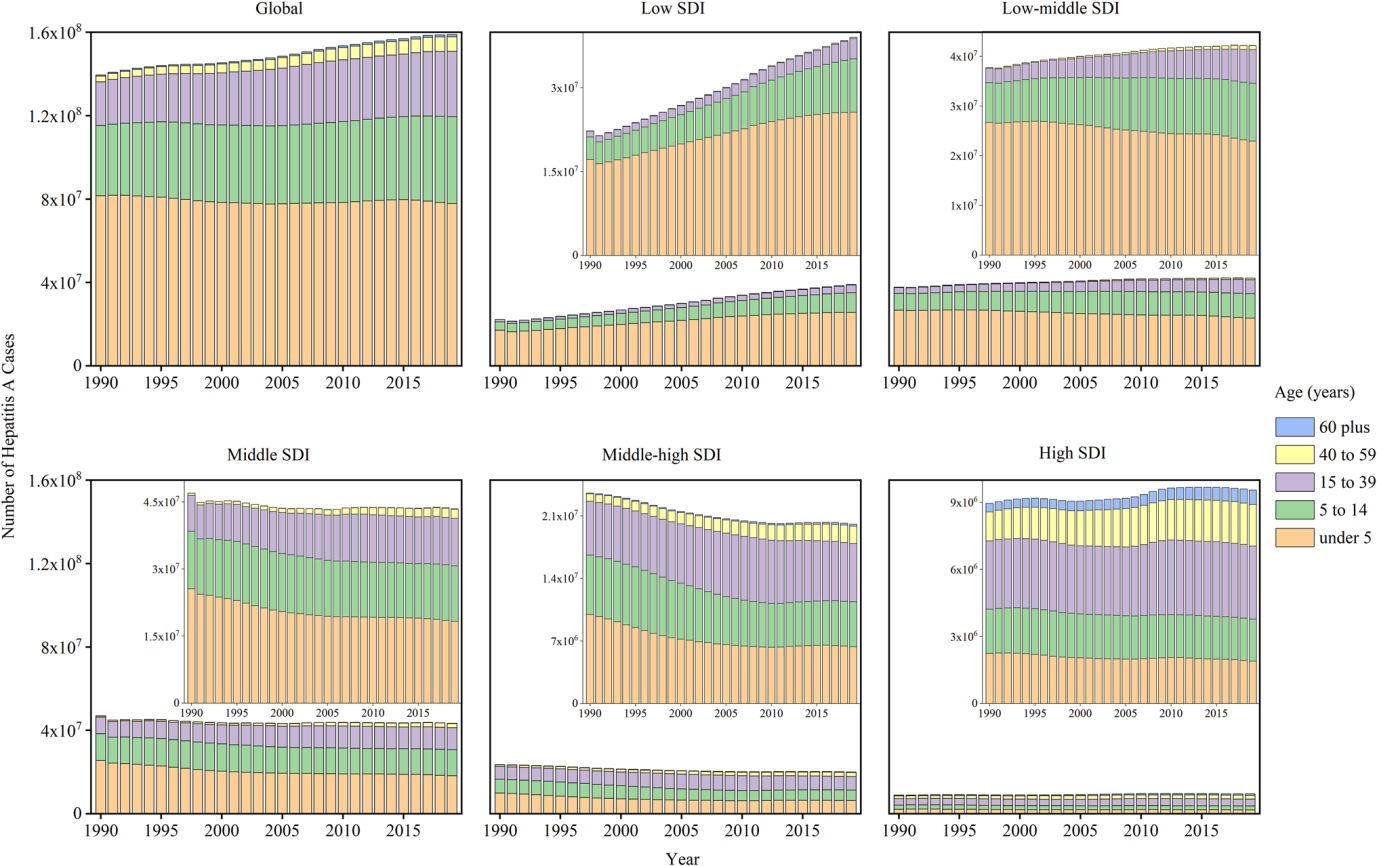
The number of incident cases of hepatitis A by age group, by SDI regions, from 1990 to 2019. The data of five SDI regions are presented in the top-right panel. *SDI* socio-demographic index

Figure 3 shows the number of incidents cases of hepatitis A by age at 5 SDI regions from 1990 to 2019. In low SDI regions, the growing number of incident cases of hepatitis A was due to the gradual increase in the number of cases in all age groups, especially in children aged under 5 years (Fig. 3A). The increased incidents cases of children aged under 5 years accounted for 50.16% of the total increased incident cases from 1990 to 2019. However, the number of incident cases was deceasing only in children aged under 5 years but increasing in other age groups in low-middle SDI regions. In middle and above SDI regions (including middle, middle-high, and high SDI regions), the number of incident cases was decreasing in children aged less than 15 years but increasing in population aged 15 years plus.

### Trends in incidence and mortality rates of hepatitis A across 21 GBD regions

For the 21 GBD regions, the incident cases of hepatitis A increased even more in GBD regions with larger population increases, such as Western Sub-Saharan Africa (110.95%), Oceania (107.85%), Central Sub-Saharan Africa (98.94%), and Eastern Sub-Saharan Africa (85.71%) (Table 1). Low-income regions suffered the severest threat of hepatitis A, with the highest ASIR of 2625.34 per 100,000 in Eastern Sub-Saharan Africa, followed by Western Sub-Saharan Africa (2625.34 per 100,000 in 2019) and Central Sub-Saharan Africa (2543.07 per 100,000 in 2019) in 2019 (Table 2). The trends in ASIRs of hepatitis A were heterogeneous across the 21 GBD regions, with the highest increasing trend in high-income Asia Pacific (EAPC = 0.53; 95% CI 0.41–0.66), Oceania (EAPC = 0.31; 95% CI 0.25–0.36), and Australasia (EAPC = 0.28; 95% CI 0.13–0.44); and stable in Western Europe, Central Latin America, North Africa and Middle East, and Western Sub-Saharan Africa. Nearly all middle-income regions had a decreased trend in ASIRs of hepatitis A, such as Central Asia, East Asia, and Southeast Asia (Table 2). The ASMR decreased in all 21 GBD regions, with the largest decrease in middle-income regions, such as East Asia (EAPC = −12.76; 95% CI −13.47 to −12.04), Central Europe (EAPC = −10.88; 95% CI −11.80 to −9.94), and Southern Latin America (EAPC = −8.65; 95% CI −9.32 to −7.98) (Table 2).

**Fig. 4:**
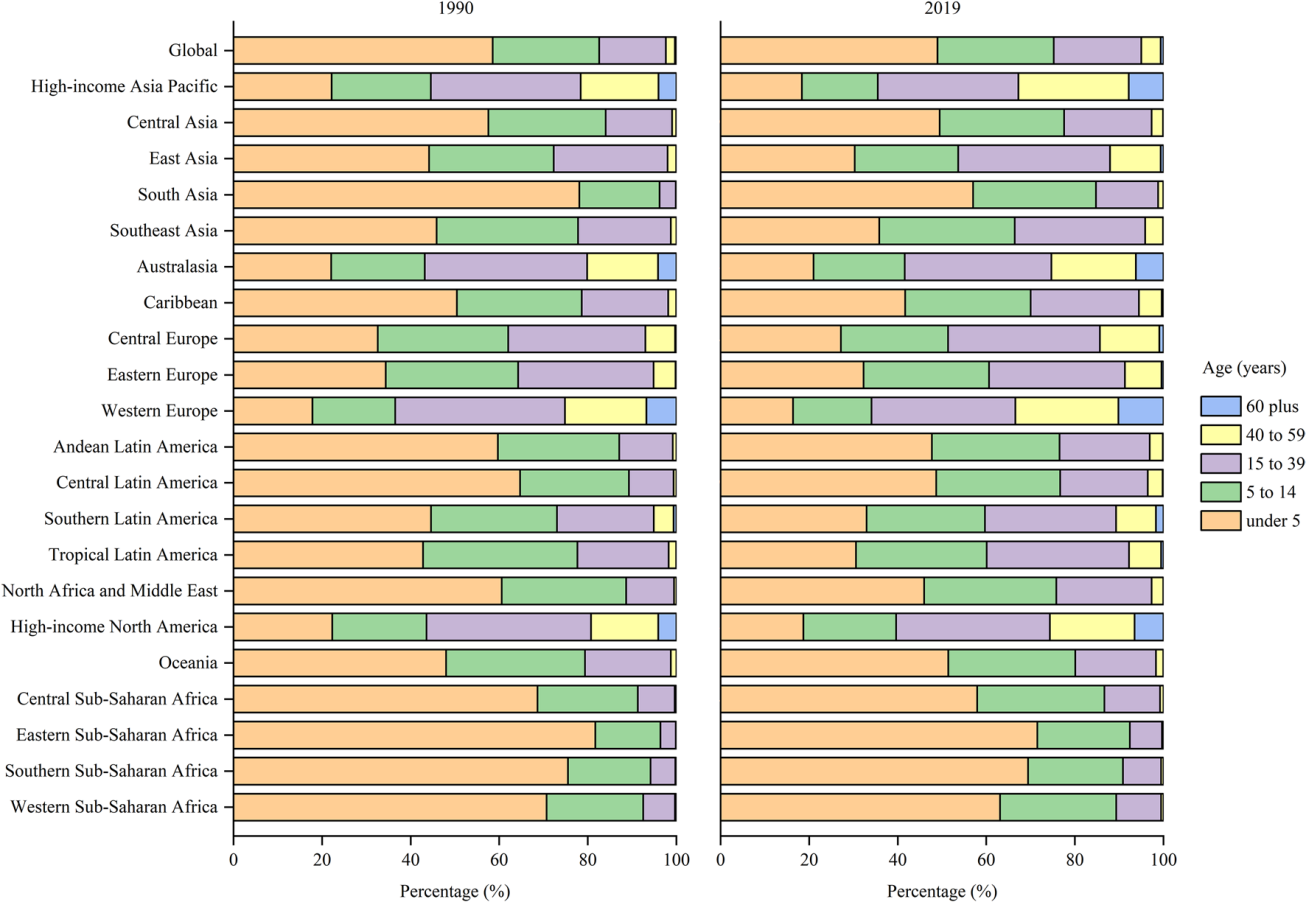
The age distribution of incident cases of hepatitis A by GBD region in 1990 and 2019. *GBD* Global Burden of Disease

Figure 4 shows the proportions of incident cases of hepatitis A by age at 21 GBD regions in 1990 and 2019. The children aged under 5 years accounted for approximately 50% incident cases globally, and even about 70% in some low-income regions, including Eastern Sub-Saharan Africa and Southern Sub-Saharan Africa. All high-income regions, including high-income Asia Pacific, Australasia, Western Europe and high-income North America, had a much lower proportions of incident cases among children aged under 5 which was less than 20%. However, adults aged 15–39 years had the highest proportions of incident cases in these high-income regions. In addition, the proportions of incident cases were high in older adults aged 60 years and older in these regions, even 10.18% in Western European.

### Factors associated with EAPC of ASIR

**Fig. 5:**
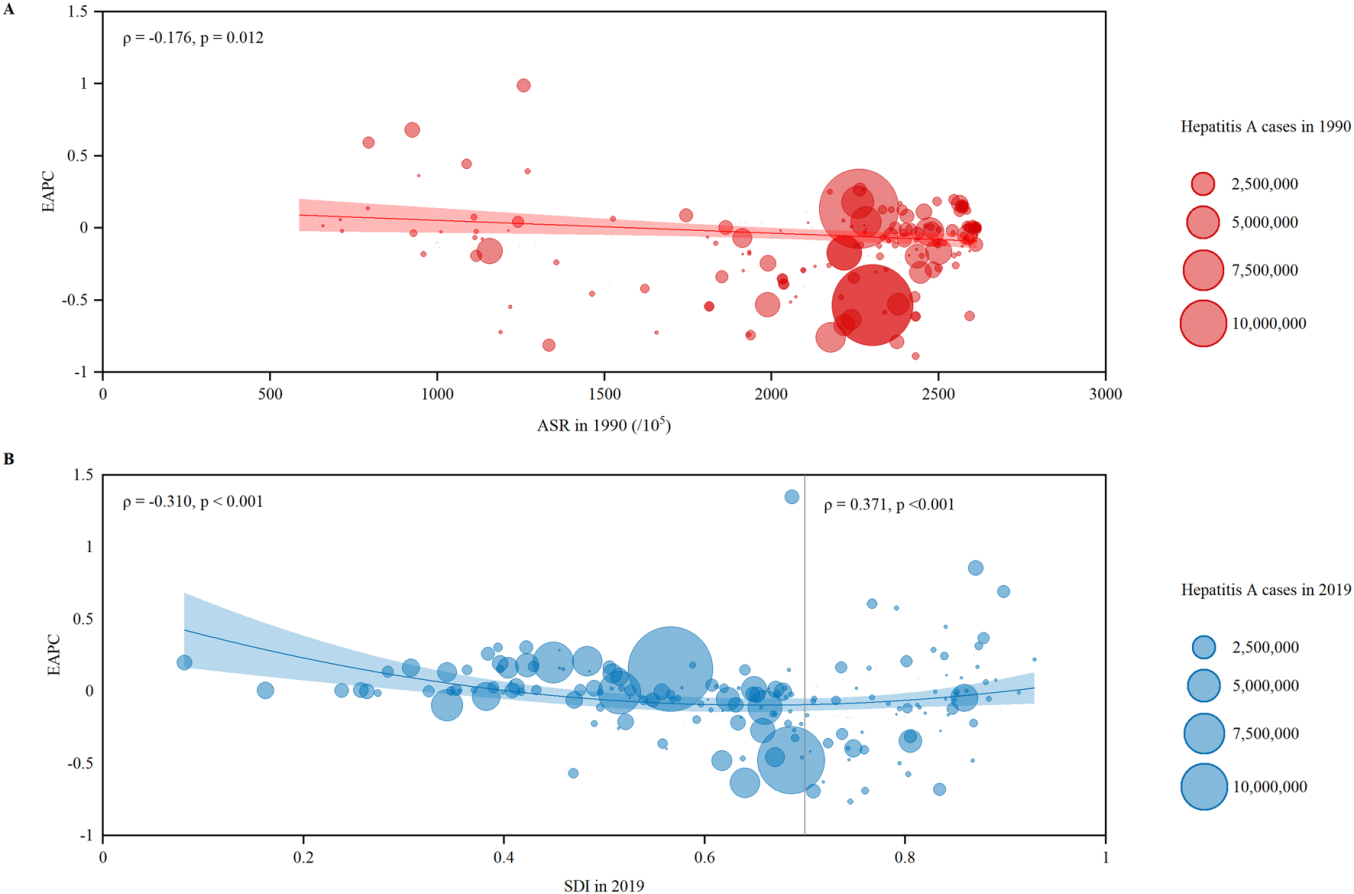
The EAPCs of hepatitis A ASIRs at countries and territorial level. **A** The correlation between EAPC and ASIR of hepatitis A in 1990. **B** The correlation between EAPC and SDI in 2019. The incident cases of hepatitis A from 204 countries and territories were represented by the circles. The size of circles increased with the incident cases of hepatitis A. The *ρ* indices and *p* values were derived from Pearson correlation analysis. *ASIR* age-standardized incidence rate, *EAPC* estimated annual percentage change, *SDI* socio-demographic index

A significant negative correlation was observed between EAPC and ASIR in 1990 (*ρ* = −0.176, *p* = 0.012) (Fig. 5A). Surprisingly, a significant positive correlation was detected between EAPC and SDI (*ρ* = 0.371, *p* < 0.001) for the countries and territories with SDI value ≥ 0.7, while a significant negative correlation was observed in the countries and territories with SDI value < 0.7 (*ρ* = −0.310, *p* < 0.001) (Fig. 5B). For SDI value ≥ 0.7, countries or territories with higher SDI have experienced an increased trend in ASIR from 1990 to 2019.

## Discussion

This study assessed the global landscape, long-term trends, and regional differences in incidence and mortality of hepatitis A using a post hoc analysis of data from the GBD study 2019. Globally, the ASIR of hepatitis A remained stable from 1990 to 2019, while the number of incident cases has increased by 13.90% in this period. Both the number of deaths and ASMR of hepatitis A decreased from 1990 to 2019. Of note, hepatitis A is not only still pending in hyperendemic regions but also emerging in low endemic regions. For example, low-income regions suffered the severest threat of hepatitis A, with the highest ASIR of hepatitis A in Eastern Sub-Saharan Africa, Western Sub-Saharan Africa, and Central Sub-Saharan Africa in 2019. In low and low-middle SDI regions, not only the ASIR of hepatitis A has increased, but the number of incident cases has also remarkably increased from 1990 to 2019. In addition, high-income Asia Pacific, Oceania, and Australasia had the highest increasing trend in ASIR of hepatitis A from 1990 to 2019. Therefore, facing with the unfavorable trend in ASIR of hepatitis A, novel challenges were posed for controlling or eliminating this disease.

This study found that the incident cases of hepatitis A increased globally between 1990 and 2019. The increased incident cases were primarily driven by population growth across the globe, specifically in low-income and middle-income countries. China and India with the world’s largest population, over 2.8 billion, accounting for nearly 40% of the earth’s inhabitants, had approximately one third of hepatitis A incident cases of the global in 2019 [33]. Moreover, immigrant growth may also play an important role in driving the increase of incident cases of hepatitis A. For example, we found the largest increase of incident cases of hepatitis A in Qatar where large numbers of immigrant workers often from low-income countries have been drawn in the last 2 decades [34]. In the growing population of immigrant families, pediatric travelers could be silent reservoirs of hepatitis A after return from the visiting of friends and relatives travel [35]. Finally, the number of hepatitis A cases could increase with the globalization processes and increased high-risk populations of hepatitis A [24, 28].

Consistent with previous studies [29, 36], this study found the incidence of hepatitis A being closely related to socioeconomic development. We found that hepatitis A tended to be more prevalent in low-income regions, including Sub-Saharan Africa and South Asia, where HAV is highly endemic and most persons become infected in early childhood [29]. Poor sanitary and hygienic conditions contributed to the propagation of HAV in low-income regions, including household crowding, poor levels of sanitation and inadequate water supplies [29, 37]. In addition, we found a decreased trend in incidence of hepatitis A in most middle-income regions, but an increased trend in Andean Latin America and Oceania. In middle-income regions, despite sanitary and hygienic conditions varying, nearly all populations have access to clean water and some children during their early childhood can avoid HAV infection, especially in urban areas [24, 29]. In this study, we found a lower proportions of incident cases in children aged under 5 years in middle-income regions than low-income regions. Finally, we found that the trends in incidence of hepatitis A increased in two high-income regions, including high-income Asia Pacific and Australasia. Good sanitation and hygienic conditions are available and infection rates of hepatitis A in children are generally low in most high-income countries [29, 37]. However, most adults are still susceptible to HAV infection and foodborne outbreaks of hepatitis A are becoming more frequent in high-income regions [24]. The peak rates of HAV infection tend to occur in adolescents and young adults. In addition, the burden of hepatitis A still significantly attribute to large community-wide outbreaks with person-to-person transmission. Finally, HAV infection predominates among persons at high risk in these high-income regions, such as travelers to countries with high endemicity levels of hepatitis A, MSM, PWIDs, and homeless persons [28, 29, 37, 38].

We found that the trends in incidence of hepatitis A decreased in most countries and territories, such as Belarus, China, and Brazil. In these countries, rising socioeconomic levels and increased access to good sanitation and hygiene facilities and clean drinking water contributed to the decreased incidence of hepatitis A [24, 36, 39]. In addition, the availability of a hepatitis A vaccine that was developed in the 1990s also played an important role in reducing this disease incidence [17, 37, 40]. For example, a shellfish-associated hepatitis A outbreak caused 300,000 cases in Shanghai, China, in two months of the first quarter of 1988 [41]. With the very rapid socioeconomic development, improved sanitary and hygienic conditions, and the introduction of hepatitis A vaccine in China, the incidence rate of hepatitis A dropped dramatically in the following couple of decades [42–44]. In addition, the decreased incidence of hepatitis A in China is also driving a decreased trend in ASIRs of hepatitis A in East Asia since China is the most populous country in East Asia. Despite the above effective ways combating hepatitis A being available for several decades, the incidence of hepatitis A still increased in high endemic countries and even some low endemic countries, such as Japan, Germany, Canada, and Italy. In countries with high endemicity levels of hepatitis A, HAV is wide spread due to poor sanitary conditions and hygienic practices, leading an increased trend in incidence of hepatitis A [24]. In the countries where the risk of HAV infection from food or water is low, there were hepatitis A outbreaks among population at high risk of hepatitis A [23, 45–50]. For example, hepatitis A outbreaks were particularly driven by transmission between non-immune MSM engaging in high-risk sexual behavior, which has been reported in European and United States populations [23, 46–49]. In addition, hepatitis A outbreaks often occurred among PWIDs, and homelessness in United States [23, 50]. Finally, globalization have driven increased international trade, migration and travel which are expected to increase foodborne outbreaks of hepatitis A in high-income countries [7, 24]. In the last decades, imported frozen berries have caused numerous foodborne outbreaks of hepatitis A in Australia and Europe [25, 51, 52]. Hepatitis A outbreaks usually occurred when residents in low endemic countries migrated and traveled to areas with high endemicity levels of HAV [24]. Evidence has reported that the hepatitis A outbreaks in Hispanic children in urban areas of the United States–Mexico border were significantly associated with cross-border travel from the United States to Mexico and foodborne exposures during the travel [53]. Therefore, facing with the complex situation of hepatitis A, more efforts should be made to eliminate hepatitis A infection globally.

In this study, a faster decreasing temporal trend in ASIR of hepatitis A between 1990 and 2019 was observed in the country with a higher baseline ASIR in 1990. It was probably because the country with higher baseline ASIR was easier to reduce the massive hepatitis A outbreaks by the development of countries’ economies and public health infrastructure, as well as the introduce of hepatitis A vaccine [24]. Furthermore, we found that in the countries or territories with SDI value ≥ 0.7, those with higher SDI have experienced an increased trend in ASIR of hepatitis A from 1990 to 2019. The possible explanations might be that the increasing age at midpoint of population immunity could increase the hepatitis A cases in high-risk populations which is more likely in high SDI countries [7]. Thus, population aging in high SDI countries could increase adults susceptible to HAV infection, leading an increased trend in hepatitis A cases and ASIR of hepatitis A in these countries [54]. In addition, outbreaks of hepatitis A among MSM have been reported in most high SDI countries in recent years, which could play an important role in increased trend in ASIR of hepatitis A in these countries [27, 55].

Although the globalization processes and increased high-risk populations of hepatitis A possibly derived an increased trend in ASIR of hepatitis A in several regions, such as high-income Asia Pacific, Oceania and Australasia, all regions have shown varying degrees of a decreased trend in ASMR of hepatitis A in this study. The decreased trend in ASMR of hepatitis A mainly benefits from vaccination strategy, increases in the quality of health care, and greater availability of liver transplantation [17, 37, 56, 57]. The globally uneven distribution of these factors mentioned above may explain the discrepancy of decreased trend in ASMR of hepatitis A across regions [58–60]. There is no specific treatment for HAV infection and treatment relies on supportive management, underscoring the importance of prevention. Hepatitis A vaccine can effectively prevent HAV infection in people at high mortality risk of hepatitis A, such as older adults and patients with chronic liver diseases [17, 37]. HAV infection causes usually self-limiting hepatitis but rarely fulminant liver failure (< 1% of cases). However, fulminant liver failure from hepatitis A may rapidly progress within a week, often resulting in death or an emergent liver transplant [61, 62]. Thus, early diagnose of hepatitis A-associated fulminant liver failure and urgent decision on liver transplantation are required [63–65]. Previous studies have reported that patients with hepatitis A-associated fulminant liver failure showed that 55–57% of patients spontaneously recovered, 31–38% underwent liver transplantation, and 6–14% died without transplantation [64, 65]. Survival rates of 65% or greater may be achieved by early referral for liver transplantation in patients with cerebral edema and multiple organ failure [3].

Since 1999, several countries have introduced universal HAV vaccination of children against HAV obtained high HAV vaccination coverage across these countries [59]. For instance, despite slow uptake in the first three years universal HAV vaccination program introduction in Israel, HAV vaccination coverage reached about 90% between 2003 and 2011 [59, 66]. In Greece, HAV vaccination coverage in 6-year-old children varied from 12.4 to 49.4% across different regions before universal HAV vaccination implementation and obtained more than 80% within 4 years post-introduction universal HAV vaccination [67]. Previous studies reported that anti-HAV antibody persistence was up to 15 years post-vaccination in ≥ 90% of vaccinated children [59, 68]. In adults, anti-HAV antibody persistence was up to 20 years post-vaccination and seropositivity rates could be ≥ 90% for up to 40 years and ≥ 85% for up to 50 years post-vaccination according to a mathematical modeling [69].

The risk of hepatitis A outbreaks can persist for extended time periods in unvaccinated individuals [24]. Thus, prevention measures that can reduce the risk for transmission of HAV should be emphasized. Sanitation measures play an important role in HAV infection prevention, including but not limited to careful attention to hygiene, particularly in the food service industry [35]. Heating foods to 185 °F (85 °C) for 1 min, use of a 1:100 solution of household bleach, handwashing, and avoiding contact with uncooked foods, are all techniques that may reasonably decrease the likelihood of hepatitis A transmission [70]. In addition, prophylaxis of vulnerable populations and children through active or passive immunization is the most important prevention practice [17, 71, 72]. WHO recommends that hepatitis A vaccine be integrated into the national immunization schedule for children aged 1 year and older in countries where clinical hepatitis A is an important health problem and immunization is likely to be a cost-effective public health tool to control this disease [73]. The recommendations for vaccination varied by the countries’ endemicity of hepatitis A. In high endemic countries, large-scale immunization efforts against hepatitis A should not be undertaken due to near universal immunity from asymptomatic childhood infections. Nationwide vaccination might be considered in intermediate endemic countries where the transmission of HAV occurs primarily from person to person in the general community. In low endemic countries, vaccination is considered for high-risk groups, such as MSM, PWIDs, homelessness and persons traveling to high-risk areas. The comprehensive plan for prevention and control of viral hepatitis should take surveillance and vaccination against hepatitis A into its part.

Despite vaccines against HAV can prevent hepatitis A, they are futile for hepatitis A-associated fulminant hepatitis [74–76]. In addition, hepatitis A patients had increased risk of comorbidities such as liver disease, disorders of lipid metabolism and chronic kidney disease, leading to prolonged hospitalization [75]. Therefore, anti-HAV drug and therapeutic intervention strategies for preventing progression to a life-threatening liver disease from hepatitis A are still in need. Previous studies have reported that small interfering RNAs can silence translation and replication of the firefly luciferase-encoding HAV replicon and is a most promising candidate for future therapeutic application to cure severe or fulminant cases of hepatitis A [74, 77]. Moreover, evidence suggests that HAV internal ribosomal entry-site (IRES) is an attractive target of antiviral agents against HAV and the JAK2 inhibitor AZD1480 can inhibit IRES activity and HAV replication [75, 76]. Recently, Japanese miso extracts have been reported to have inhibitory effects on HAV replication not only in patients infected with HAV but also in patients superinfected with HAV and HBV [9].

The GBD study provides a better understanding of the trend in incidence of hepatitis A over the last couple of decades globally. Some limitations in this study should also be acknowledged. First, the accuracy and robustness of GBD estimates largely depend on the quality and quantity of data used in the modeling [32]. Second, due to the GBD study taking the country as its basic unit, the incidence of hepatitis A might be a margin of bias in countries in lack of national systematic surveillance and population-based studies of hepatitis A. Third, although the standardization makes the incidence and mortality rates of hepatitis A comparable at the global, regional and national levels, the ASIR and ASMR of hepatitis A only reflect the burden of hepatitis A for one region or country under the age structure of a GBD World Standard Population.

## Conclusion

In summary, there is an unfavorable trend in ASIR of hepatitis A that it is still pending in hyperendemic regions and is emerging in low endemic regions globally. Due to the rapid globalization processes and increased high-risk populations of hepatitis A, the number of hepatitis A cases is probably in an increasing trend, posing novel challenges for eliminating this disease, especially in high-income regions. Thus, more targeted and specific strategies were needed to eliminate hepatitis A, such as sanitation measures and a comprehensive plan for surveillance and vaccination against hepatitis A. Future studies focusing on targeted prevention and control strategies for hepatitis A were warranted.

## Supplementary Information

Below is the link to the electronic supplementary material.

Supplementary file1 (DOCX 50 KB)

## Data Availability

All data were obtained from the Global Health Data Exchange (GHDx) query tool (http://ghdx.healthdata.org/gbd-results-tool) and the United Nations, Department of Economic and Social Affairs, Population Division (2019) (https://population.un.org/wpp/).
