# Supplementary material for: The global trends and regional differences in incidence and mortality of hepatitis A from 1990 to 2019 and implications for its prevention
Source: Hepatol Int. 2021 Aug 3;15(5):1068–82. doi: 10.1007/s12072-021-10232-4 (PMC8514357; doi:10.1007/s12072-021-10232-4)
Supplement: Supplementary file 1 — Supplementary file1 (DOCX 50 KB) [file 12072_2021_10232_MOESM1_ESM.docx]

**Table S1**. The detailed list of 21 GBD regions.

| Regions | GBD regions |
| --- | --- |
| Low income regions | South Asia, Central Sub-Saharan Africa, Eastern Sub-Saharan Africa, Southern Sub-Saharan Africa, Western Sub-Saharan Africa |
| Middle-income regions | Central Asia, East Asia, Southeast Asia, Caribbean, Central Europe, Eastern Europe, Andean Latin America, Central Latin America, Southern Latin America, Tropical Latin America, North Africa and Middle East, Oceania |
| High-income regions | High-income Asia Pacific, Australasia, Western Europe, High-income North America |

GBD: Global Burden of Disease.

**Table S2**. The SDI value of 204 countries and territories in 2019, and the incident cases and ASIRs of hepatitis A in 1990 and 2019 and their temporal trends from 1990 to 2019 at national level.

| Region | 1990 | |  | 2019 | |  | 1990-2019 | |  | SDI  value  in 2019 |
| --- | --- | --- | --- | --- | --- | --- | --- | --- | --- | --- |
|  | Incident cases  No. x 10^3^ (95% UI) | ASIR per 100,000  No. (95% UI) |  | Incident cases  No. x 10^3^ (95% UI) | ASIR per 100,000  No. (95% UI) |  | Percentage change  of incident cases  (%) | EAPC  No. (95% CI) |  |  |
| Afghanistan | 435.57 (395.52, 478.36) | 2,302.10 (2,114.90, 2,487.53) |  | 1,509.03 (1,382.20, 1,635.62) | 1,961.38 (1,758.94, 2,157.18) |  | 246.45 | -0.48 (-0.54, -0.42) |  | 0.343 |
| Albania | 94.78 (85.11, 103.64) | 2,035.63 (1,802.02, 2,242.73) |  | 46.47 (43.00, 49.83) | 1,836.18 (1,611.41, 2,058.13) |  | -50.97 | -0.36 (-0.39, -0.34) |  | 0.681 |
| Algeria | 858.01 (789.48, 925.63) | 1,813.23 (1,604.40, 1,998.17) |  | 996.49 (916.21, 1,074.91) | 1,708.28 (1,507.90, 1,910.82) |  | 16.14 | -0.22 (-0.54, 0.10) |  | 0.652 |
| American Samoa | 1.27 (1.13, 1.42) | 2,430.40 (2,229.11, 2,626.56) |  | 1.12 (1.00, 1.23) | 2,100.28 (1,888.33, 2,315.69) |  | -11.32 | -0.57 (-0.61, -0.52) |  | 0.712 |
| Andorra | 0.48 (0.43, 0.53) | 2,217.55 (2,018.46, 2,410.67) |  | 0.64 (0.59, 0.69) | 2,128.83 (1,943.33, 2,321.57) |  | 32.75 | -0.11 (-0.17, -0.05) |  | 0.894 |
| Angola | 457.72 (421.29, 492.80) | 2,094.37 (1,880.26, 2,309.02) |  | 1,188.89 (1,087.89, 1,282.14) | 1,966.49 (1,750.88, 2,175.42) |  | 159.74 | -0.22 (-0.29, -0.15) |  | 0.470 |
| Antigua and Barbuda | 1.55 (1.42, 1.66) | 2,032.73 (1,824.36, 2,234.11) |  | 1.50 (1.38, 1.61) | 1,819.57 (1,615.07, 2,024.17) |  | -2.81 | -0.29 (-0.35, -0.24) |  | 0.743 |
| Argentina | 813.48 (714.07, 895.26) | 2,097.96 (1,893.69, 2,320.20) |  | 814.99 (728.42, 900.49) | 1,873.65 (1,662.75, 2,074.01) |  | 0.18 | -0.40 (-0.41, -0.39) |  | 0.708 |
| Armenia | 86.66 (78.14, 94.51) | 1,989.34 (1,784.96, 2,192.79) |  | 50.55 (45.85, 54.97) | 1,873.22 (1,667.68, 2,067.44) |  | -41.67 | -0.21 (-0.24, -0.18) |  | 0.689 |
| Australia | 171.80 (152.41, 191.97) | 1,911.89 (1,730.99, 2,100.25) |  | 240.46 (212.46, 265.88) | 1,878.34 (1,689.78, 2,063.28) |  | 39.96 | -0.06 (-0.07, -0.04) |  | 0.839 |
| Austria | 72.92 (65.28, 81.03) | 1,620.45 (1,436.55, 1,798.08) |  | 75.88 (68.67, 83.74) | 1,473.46 (1,305.65, 1,660.36) |  | 4.06 | -0.32 (-0.42, -0.23) |  | 0.849 |
| Azerbaijan | 222.02 (204.23, 238.87) | 1,258.83 (1,141.45, 1,380.71) |  | 207.19 (191.62, 222.11) | 1,688.37 (1,499.63, 1,871.03) |  | -6.68 | 1.35 (0.99, 1.71) |  | 0.683 |
| Bahamas | 6.43 (5.93, 6.94) | 2,130.00 (1,915.66, 2,348.14) |  | 6.60 (6.06, 7.07) | 1,988.90 (1,766.84, 2,199.97) |  | 2.69 | -0.26 (-0.27, -0.24) |  | 0.796 |
| Bahrain | 13.72 (12.51, 14.88) | 2,379.16 (2,166.50, 2,588.93) |  | 21.84 (20.33, 23.28) | 2,091.70 (1,861.67, 2,321.41) |  | 59.18 | -0.48 (-0.53, -0.43) |  | 0.751 |
| Bangladesh | 3,984.43 (3,677.67, 4,307.05) | 2,027.07 (1,830.63, 2,230.20) |  | 3,670.92 (3,427.45, 3,931.71) | 2,034.97 (1,823.04, 2,247.47) |  | -7.87 | 0.02 (-0.02, 0.05) |  | 0.483 |
| Barbados | 5.22 (4.82, 5.62) | 2,119.99 (1,913.25, 2,333.65) |  | 4.32 (3.95, 4.64) | 2,142.31 (1,923.11, 2,338.21) |  | -17.22 | 0.04 (0.01, 0.07) |  | 0.742 |
| Belarus | 211.71 (193.69, 230.04) | 2,461.57 (2,257.92, 2,651.76) |  | 131.51 (118.86, 143.32) | 2,455.75 (2,250.16, 2,665.67) |  | -37.88 | 0.00 (-0.02, 0.02) |  | 0.745 |
| Belgium | 94.61 (87.65, 102.09) | 1,514.37 (1,349.99, 1,695.98) |  | 100.51 (90.63, 111.39) | 1,399.35 (1,250.47, 1,558.40) |  | 6.23 | -0.22 (-0.38, -0.07) |  | 0.851 |
| Belize | 6.60 (6.05, 7.14) | 2,174.20 (1,969.23, 2,376.53) |  | 10.13 (9.36, 10.88) | 2,365.96 (2,146.92, 2,574.65) |  | 53.61 | 0.31 (0.25, 0.36) |  | 0.603 |
| Benin | 235.12 (217.87, 251.66) | 1,904.49 (1,706.63, 2,092.09) |  | 534.76 (494.17, 571.90) | 1,946.63 (1,739.11, 2,147.50) |  | 127.44 | 0.06 (0.01, 0.11) |  | 0.352 |
| Bermuda | 1.09 (1.00, 1.17) | 2,109.32 (1,899.21, 2,314.39) |  | 0.80 (0.74, 0.85) | 2,141.33 (1,930.50, 2,343.44) |  | -26.61 | 0.07 (0.04, 0.10) |  | 0.813 |
| Bhutan | 21.19 (19.54, 22.95) | 2,020.06 (1,823.21, 2,224.81) |  | 17.03 (15.66, 18.37) | 2,016.01 (1,807.14, 2,222.28) |  | -19.63 | -0.02 (-0.07, 0.03) |  | 0.455 |
| Bolivia | 243.98 (226.12, 262.80) | 2,104.28 (1,902.79, 2,314.43) |  | 347.44 (318.65, 375.54) | 2,185.25 (1,970.14, 2,396.07) |  | 42.40 | 0.14 (0.11, 0.17) |  | 0.566 |
| Bosnia and Herzegovina | 80.26 (72.06, 88.03) | 2,342.81 (2,117.65, 2,549.25) |  | 35.80 (32.34, 39.36) | 2,190.52 (1,964.69, 2,412.44) |  | -55.39 | -0.27 (-0.29, -0.25) |  | 0.718 |
| Botswana | 50.03 (46.21, 53.67) | 2,550.00 (2,357.95, 2,741.36) |  | 63.51 (58.16, 68.24) | 2,411.86 (2,202.40, 2,614.31) |  | 26.95 | -0.22 (-0.26, -0.19) |  | 0.634 |
| Brazil | 3,813.38 (3,523.61, 4,122.62) | 2,449.53 (2,246.44, 2,636.02) |  | 3,692.94 (3,371.23, 3,983.75) | 2,361.35 (2,131.93, 2,573.48) |  | -3.16 | -0.17 (-0.19, -0.14) |  | 0.640 |
| Brunei | 3.93 (3.37, 4.50) | 2,427.56 (2,208.12, 2,618.06) |  | 5.10 (4.49, 5.70) | 2,183.61 (1,939.99, 2,402.89) |  | 29.75 | -0.36 (-0.48, -0.24) |  | 0.823 |
| Bulgaria | 149.37 (136.91, 161.61) | 2,513.50 (2,320.76, 2,703.75) |  | 94.99 (87.76, 101.47) | 2,458.54 (2,246.04, 2,653.06) |  | -36.41 | -0.09 (-0.10, -0.07) |  | 0.764 |
| Burkina Faso | 449.73 (418.32, 482.04) | 2,560.24 (2,381.09, 2,748.82) |  | 973.06 (902.34, 1,040.93) | 2,466.38 (2,259.29, 2,656.69) |  | 116.37 | -0.13 (-0.18, -0.08) |  | 0.257 |
| Burundi | 267.97 (249.41, 286.15) | 2,580.87 (2,391.07, 2,774.26) |  | 522.16 (485.88, 556.28) | 2,575.53 (2,391.82, 2,770.93) |  | 94.86 | -0.06 (-0.09, -0.04) |  | 0.284 |
| Cambodia | 402.89 (364.31, 441.63) | 2,545.70 (2,350.03, 2,736.85) |  | 374.93 (337.49, 413.19) | 2,443.46 (2,233.60, 2,645.18) |  | -6.94 | -0.16 (-0.19, -0.12) |  | 0.469 |
| Cameroon | 469.05 (438.75, 499.57) | 2,609.89 (2,431.13, 2,789.59) |  | 1,067.18 (992.75, 1,138.00) | 2,582.09 (2,389.80, 2,772.87) |  | 127.52 | -0.09 (-0.11, -0.07) |  | 0.490 |
| Canada | 231.64 (206.59, 257.50) | 2,432.17 (2,196.99, 2,647.35) |  | 274.55 (249.31, 299.17) | 2,466.16 (2,249.00, 2,665.62) |  | 18.52 | -0.04 (-0.08, 0.01) |  | 0.873 |
| Cape Verde | 14.18 (13.18, 15.13) | 1,932.14 (1,711.82, 2,140.22) |  | 14.38 (13.40, 15.30) | 1,681.46 (1,480.91, 1,889.22) |  | 1.42 | -0.63 (-0.74, -0.52) |  | 0.525 |
| Central African Republic | 117.15 (108.07, 125.92) | 2,357.98 (2,130.70, 2,588.87) |  | 201.60 (185.42, 216.75) | 2,477.71 (2,243.67, 2,695.97) |  | 72.09 | 0.16 (0.13, 0.19) |  | 0.274 |
| Chad | 292.75 (270.68, 314.49) | 1,655.17 (1,462.42, 1,854.49) |  | 790.23 (731.80, 846.99) | 1,558.09 (1,352.27, 1,770.12) |  | 169.94 | -0.50 (-0.73, -0.27) |  | 0.238 |
| Chile | 371.30 (343.54, 397.63) | 1,525.67 (1,337.19, 1,710.11) |  | 312.77 (283.45, 340.48) | 1,417.40 (1,244.51, 1,582.13) |  | -15.76 | 0.29 (0.06, 0.51) |  | 0.759 |
| China | 27,889.16 (25,747.92, 29,996.19) | 1,269.97 (1,115.00, 1,422.33) |  | 19,369.25 (17,669.96, 20,975.47) | 1,323.51 (1,154.81, 1,483.64) |  | -30.55 | 0.58 (0.39, 0.76) |  | 0.686 |
| Colombia | 1,019.64 (939.31, 1,099.15) | 2,057.35 (1,825.93, 2,279.27) |  | 985.31 (909.00, 1,059.81) | 1,829.19 (1,617.94, 2,054.38) |  | -3.37 | -0.48 (-0.51, -0.44) |  | 0.633 |
| Comoros | 20.52 (19.08, 21.92) | 1,913.96 (1,681.06, 2,134.11) |  | 21.40 (19.73, 22.83) | 1,852.22 (1,628.57, 2,066.34) |  | 4.27 | -0.16 (-0.18, -0.14) |  | 0.455 |
| Congo | 100.90 (93.42, 109.31) | 1,851.68 (1,644.45, 2,047.46) |  | 169.98 (156.90, 183.52) | 1,641.78 (1,449.90, 1,838.13) |  | 68.47 | -0.12 (-0.34, 0.11) |  | 0.568 |
| Cook Islands | 0.43 (0.38, 0.48) | 1,937.64 (1,728.63, 2,136.41) |  | 0.29 (0.26, 0.32) | 1,626.35 (1,428.63, 1,827.22) |  | -32.19 | -0.69 (-0.74, -0.64) |  | 0.764 |
| Costa Rica | 95.15 (87.20, 102.45) | 2,267.15 (2,018.58, 2,503.24) |  | 96.01 (89.59, 103.21) | 2,242.16 (1,992.79, 2,467.85) |  | 0.90 | -0.04 (-0.12, 0.03) |  | 0.680 |
| Cote d'Ivoire | 558.31 (516.09, 598.95) | 1,833.07 (1,623.26, 2,049.09) |  | 985.87 (915.04, 1,057.96) | 1,778.77 (1,566.41, 1,995.02) |  | 76.58 | -0.09 (-0.11, -0.08) |  | 0.408 |
| Croatia | 64.19 (57.55, 70.60) | 1,807.43 (1,592.24, 2,029.30) |  | 42.21 (37.87, 46.64) | 1,767.04 (1,554.73, 1,980.92) |  | -34.24 | -0.05 (-0.06, -0.04) |  | 0.794 |
| Cuba | 228.77 (211.98, 245.91) | 2,431.19 (2,201.20, 2,669.92) |  | 163.63 (149.52, 174.58) | 2,022.35 (1,802.94, 2,230.40) |  | -28.47 | -0.76 (-0.89, -0.64) |  | 0.668 |
| Cyprus | 9.01 (7.77, 10.38) | 1,915.35 (1,693.81, 2,130.55) |  | 12.56 (11.10, 14.01) | 1,780.95 (1,576.54, 1,988.50) |  | 39.40 | -0.27 (-0.30, -0.25) |  | 0.841 |
| Czech Republic | 128.64 (114.77, 142.46) | 1,932.73 (1,715.06, 2,139.45) |  | 104.17 (93.90, 114.50) | 1,858.88 (1,639.46, 2,059.11) |  | -19.02 | -0.15 (-0.17, -0.14) |  | 0.828 |
| Democratic Republic of the Congo | 1,732.79 (1,593.04, 1,862.89) | 1,933.63 (1,716.35, 2,136.31) |  | 3,251.57 (3,002.23, 3,494.29) | 1,851.43 (1,644.33, 2,071.27) |  | 87.65 | -0.16 (-0.18, -0.15) |  | 0.382 |
| Denmark | 32.70 (29.49, 35.81) | 2,172.08 (1,958.33, 2,382.27) |  | 36.49 (33.15, 39.73) | 2,052.76 (1,810.65, 2,269.99) |  | 11.60 | -0.23 (-0.26, -0.19) |  | 0.890 |
| Djibouti | 22.37 (20.88, 23.88) | 1,988.23 (1,788.47, 2,175.92) |  | 41.05 (38.26, 43.75) | 2,056.07 (1,850.39, 2,244.99) |  | 83.50 | -0.34 (-0.53, -0.16) |  | 0.459 |
| Dominica | 1.99 (1.81, 2.15) | 1,863.08 (1,654.05, 2,043.81) |  | 1.22 (1.13, 1.31) | 1,847.89 (1,657.15, 2,040.04) |  | -38.52 | 0.17 (0.00, 0.33) |  | 0.729 |
| Dominican Republic | 243.52 (223.60, 263.16) | 1,293.43 (1,125.51, 1,465.61) |  | 268.02 (243.64, 291.55) | 1,255.14 (1,082.08, 1,419.06) |  | 10.06 | -0.08 (-0.10, -0.06) |  | 0.592 |
| Ecuador | 307.83 (280.70, 333.99) | 924.53 (806.73, 1,038.39) |  | 432.96 (401.63, 464.15) | 1,100.98 (963.17, 1,237.59) |  | 40.65 | 0.86 (0.68, 1.03) |  | 0.640 |
| Egypt | 1,982.98 (1,831.34, 2,127.61) | 1,744.66 (1,574.20, 1,935.50) |  | 2,546.49 (2,323.67, 2,763.38) | 1,587.81 (1,376.88, 1,804.78) |  | 28.42 | 0.37 (0.09, 0.65) |  | 0.658 |
| El Salvador | 186.68 (173.02, 199.99) | 1,134.13 (1,002.95, 1,267.46) |  | 153.70 (143.03, 164.95) | 1,211.83 (1,057.39, 1,364.80) |  | -17.67 | 0.00 (-0.08, 0.08) |  | 0.573 |
| Equatorial Guinea | 20.17 (18.51, 21.81) | 1,110.47 (973.92, 1,254.22) |  | 46.88 (43.02, 50.60) | 1,194.58 (1,029.46, 1,347.01) |  | 132.43 | 0.24 (0.08, 0.41) |  | 0.685 |
| Eritrea | 137.38 (128.69, 146.12) | 945.01 (831.46, 1,066.27) |  | 245.74 (227.77, 262.42) | 1,052.93 (924.24, 1,187.20) |  | 78.88 | 0.45 (0.36, 0.54) |  | 0.396 |
| Estonia | 25.07 (22.44, 27.62) | 996.78 (874.62, 1,118.87) |  | 15.53 (14.03, 16.98) | 964.02 (850.92, 1,084.37) |  | -38.06 | -0.09 (-0.10, -0.08) |  | 0.835 |
| Eswatini | 35.91 (33.31, 38.39) | 1,112.39 (975.61, 1,263.37) |  | 36.90 (34.36, 39.51) | 1,087.54 (951.21, 1,236.77) |  | 2.74 | -0.06 (-0.07, -0.06) |  | 0.577 |
| Ethiopia | 2,348.96 (2,186.84, 2,507.89) | 1,116.81 (1,015.98, 1,222.76) |  | 4,139.31 (3,826.19, 4,430.53) | 1,099.08 (958.83, 1,250.76) |  | 76.22 | 0.00 (-0.02, 0.02) |  | 0.343 |
| Federated States of Micronesia | 2.13 (1.86, 2.42) | 1,213.05 (1,039.77, 1,407.61) |  | 1.51 (1.35, 1.68) | 1,151.60 (988.24, 1,324.58) |  | -29.06 | 0.11 (-0.02, 0.24) |  | 0.580 |
| Fiji | 19.07 (17.15, 21.06) | 710.14 (626.61, 796.76) |  | 18.79 (16.84, 20.70) | 717.00 (629.91, 800.37) |  | -1.47 | 0.08 (0.06, 0.10) |  | 0.664 |
| Finland | 30.61 (27.75, 33.52) | 658.35 (583.37, 732.78) |  | 31.12 (28.51, 33.80) | 637.84 (565.53, 712.66) |  | 1.67 | 0.09 (0.02, 0.17) |  | 0.856 |
| France | 661.41 (599.96, 727.40) | 1,334.17 (1,191.36, 1,487.50) |  | 609.65 (547.22, 672.74) | 1,158.84 (1,009.64, 1,306.92) |  | -7.83 | -0.68 (-0.81, -0.55) |  | 0.834 |
| Gabon | 38.87 (35.66, 42.07) | 794.29 (710.42, 873.89) |  | 50.18 (46.19, 54.03) | 902.58 (792.40, 1,023.16) |  | 29.08 | 0.69 (0.59, 0.79) |  | 0.656 |
| Gambia | 46.31 (42.88, 49.86) | 1,356.33 (1,183.85, 1,538.05) |  | 83.15 (77.42, 89.16) | 1,296.55 (1,131.48, 1,471.57) |  | 79.56 | -0.12 (-0.24, 0.00) |  | 0.399 |
| Georgia | 119.29 (110.06, 127.64) | 587.92 (523.60, 653.05) |  | 62.90 (57.23, 68.07) | 611.08 (538.79, 683.40) |  | -47.27 | 0.25 (0.17, 0.33) |  | 0.702 |
| Germany | 558.60 (511.25, 604.24) | 1,217.53 (1,074.48, 1,381.51) |  | 611.11 (558.34, 667.23) | 1,080.50 (949.30, 1,225.39) |  | 9.40 | -0.48 (-0.55, -0.41) |  | 0.898 |
| Ghana | 643.51 (596.66, 688.22) | 1,189.31 (1,042.56, 1,344.21) |  | 1,027.58 (959.93, 1,093.91) | 1,106.60 (971.21, 1,252.33) |  | 59.68 | -0.57 (-0.72, -0.42) |  | 0.557 |
| Greece | 116.20 (104.20, 129.21) | 1,241.45 (1,121.64, 1,386.30) |  | 95.51 (86.54, 104.97) | 1,441.59 (1,279.57, 1,609.21) |  | -17.81 | 0.21 (0.04, 0.37) |  | 0.794 |
| Greenland | 0.86 (0.76, 0.97) | 1,006.73 (891.71, 1,131.60) |  | 0.72 (0.64, 0.80) | 963.48 (857.04, 1,089.17) |  | -16.83 | -0.13 (-0.14, -0.12) |  | 0.761 |
| Grenada | 2.50 (2.29, 2.71) | 1,245.75 (1,086.28, 1,396.03) |  | 1.92 (1.75, 2.06) | 1,200.58 (1,040.14, 1,364.45) |  | -23.25 | -0.10 (-0.12, -0.08) |  | 0.669 |
| Guam | 2.94 (2.62, 3.25) | 959.38 (849.16, 1,076.49) |  | 3.14 (2.79, 3.46) | 951.72 (840.03, 1,075.75) |  | 6.90 | -0.05 (-0.18, 0.08) |  | 0.813 |
| Guatemala | 350.21 (322.33, 377.52) | 1,010.36 (896.31, 1,132.88) |  | 532.68 (491.76, 573.46) | 1,005.84 (888.98, 1,131.45) |  | 52.10 | -0.01 (-0.03, 0.01) |  | 0.526 |
| Guinea | 286.67 (264.06, 307.72) | 1,462.29 (1,278.10, 1,660.96) |  | 521.86 (483.16, 559.02) | 1,247.02 (1,079.72, 1,430.84) |  | 82.04 | -0.40 (-0.46, -0.34) |  | 0.325 |
| Guinea-Bissau | 45.66 (42.29, 48.86) | 1,087.43 (1,017.38, 1,159.95) |  | 71.97 (67.17, 76.63) | 1,146.55 (1,005.65, 1,297.06) |  | 57.63 | 0.61 (0.44, 0.77) |  | 0.355 |
| Guyana | 24.05 (22.06, 26.13) | 714.62 (627.10, 805.77) |  | 17.74 (16.18, 19.22) | 654.67 (577.20, 733.44) |  | -26.24 | 0.17 (-0.02, 0.36) |  | 0.618 |
| Haiti | 230.53 (209.70, 251.53) | 792.30 (698.26, 878.85) |  | 366.13 (334.51, 396.64) | 826.97 (730.49, 921.93) |  | 58.82 | 0.22 (0.14, 0.31) |  | 0.432 |
| Honduras | 182.45 (164.75, 198.46) | 1,116.75 (990.97, 1,244.91) |  | 273.63 (248.88, 295.98) | 1,062.74 (940.92, 1,184.91) |  | 49.98 | -0.12 (-0.19, -0.05) |  | 0.496 |
| Hungary | 106.56 (95.42, 117.65) | 2,374.73 (2,088.94, 2,612.31) |  | 86.29 (77.65, 94.48) | 2,053.25 (1,817.04, 2,295.17) |  | -19.02 | -0.69 (-0.79, -0.59) |  | 0.791 |
| Iceland | 1.45 (1.29, 1.60) | 2,591.70 (2,398.23, 2,772.53) |  | 1.96 (1.77, 2.15) | 2,184.09 (1,940.03, 2,417.74) |  | 35.19 | -0.40 (-0.61, -0.20) |  | 0.869 |
| India | 26,893.65 (24,994.59, 28,990.77) | 2,337.98 (2,080.20, 2,555.69) |  | 30,385.61 (27,938.27, 32,792.21) | 2,058.52 (1,815.50, 2,279.45) |  | 12.98 | -0.46 (-0.59, -0.33) |  | 0.566 |
| Indonesia | 5,093.96 (4,661.63, 5,538.62) | 928.28 (816.24, 1,045.26) |  | 4,942.41 (4,560.94, 5,320.29) | 911.41 (802.24, 1,017.71) |  | -2.98 | 0.32 (-0.04, 0.67) |  | 0.660 |
| Iran | 1,904.96 (1,747.87, 2,069.41) | 1,156.23 (1,021.54, 1,290.83) |  | 1,623.73 (1,495.84, 1,747.22) | 1,207.25 (1,066.22, 1,350.23) |  | -14.76 | -0.05 (-0.16, 0.07) |  | 0.670 |
| Iraq | 694.41 (636.09, 753.18) | 2,351.30 (2,160.17, 2,530.78) |  | 1,153.23 (1,069.04, 1,237.09) | 2,238.68 (2,004.03, 2,451.15) |  | 66.07 | -0.18 (-0.19, -0.16) |  | 0.671 |
| Ireland | 41.94 (37.30, 47.38) | 2,302.76 (2,107.77, 2,501.42) |  | 46.16 (41.39, 51.37) | 2,197.10 (1,970.78, 2,407.46) |  | 10.06 | -0.18 (-0.21, -0.16) |  | 0.867 |
| Israel | 60.44 (52.58, 68.70) | 2,287.00 (2,081.51, 2,495.32) |  | 100.98 (88.53, 114.15) | 2,176.80 (1,949.65, 2,392.30) |  | 67.06 | -0.18 (-0.20, -0.17) |  | 0.803 |
| Italy | 574.22 (529.37, 625.63) | 2,443.89 (2,252.16, 2,636.33) |  | 581.46 (533.54, 630.78) | 2,402.31 (2,197.68, 2,603.49) |  | 1.26 | -0.06 (-0.06, -0.05) |  | 0.801 |
| Jamaica | 61.78 (56.79, 66.91) | 2,323.74 (2,123.52, 2,516.10) |  | 51.76 (47.38, 55.55) | 2,211.67 (1,967.98, 2,413.07) |  | -16.22 | -0.16 (-0.20, -0.12) |  | 0.684 |
| Japan | 994.71 (891.67, 1,094.42) | 2,387.36 (2,181.63, 2,580.10) |  | 938.30 (852.87, 1,024.24) | 2,273.16 (2,057.57, 2,483.08) |  | -5.67 | -0.17 (-0.18, -0.15) |  | 0.870 |
| Jordan | 138.20 (126.65, 150.45) | 2,499.72 (2,311.82, 2,689.70) |  | 301.02 (278.12, 324.03) | 2,429.31 (2,206.51, 2,645.60) |  | 117.81 | -0.19 (-0.28, -0.11) |  | 0.731 |
| Kazakhstan | 438.24 (398.03, 473.24) | 2,384.96 (2,189.30, 2,576.67) |  | 393.69 (350.22, 433.22) | 2,242.03 (2,012.14, 2,441.69) |  | -10.17 | -0.22 (-0.24, -0.20) |  | 0.723 |
| Kenya | 1,038.63 (966.76, 1,111.12) | 2,433.15 (2,245.93, 2,633.82) |  | 1,644.07 (1,516.78, 1,763.81) | 2,333.98 (2,114.73, 2,544.05) |  | 58.29 | -0.14 (-0.15, -0.12) |  | 0.508 |
| Kiribati | 2.13 (1.90, 2.38) | 2,476.21 (2,287.58, 2,664.75) |  | 3.03 (2.72, 3.33) | 2,480.26 (2,275.54, 2,674.47) |  | 42.21 | 0.01 (0.01, 0.02) |  | 0.527 |
| Kuwait | 46.57 (42.51, 50.44) | 2,214.19 (2,036.09, 2,395.01) |  | 79.26 (73.20, 85.07) | 2,184.01 (1,961.36, 2,382.58) |  | 70.18 | 0.15 (0.05, 0.24) |  | 0.851 |
| Kyrgyzstan | 145.62 (132.76, 157.85) | 2,377.40 (2,192.93, 2,567.01) |  | 179.63 (163.66, 194.38) | 2,235.06 (2,024.94, 2,437.65) |  | 23.36 | -0.22 (-0.24, -0.21) |  | 0.596 |
| Lao | 126.53 (112.34, 141.35) | 2,416.77 (2,235.69, 2,616.59) |  | 157.61 (140.30, 174.13) | 2,289.18 (2,071.31, 2,506.38) |  | 24.56 | -0.18 (-0.19, -0.17) |  | 0.490 |
| Latvia | 42.07 (37.72, 46.19) | 2,411.35 (2,218.67, 2,604.00) |  | 22.89 (20.60, 24.98) | 2,291.17 (2,066.14, 2,492.67) |  | -45.59 | -0.18 (-0.19, -0.17) |  | 0.820 |
| Lebanon | 102.29 (93.20, 111.43) | 2,310.73 (2,113.14, 2,494.42) |  | 112.01 (101.95, 121.72) | 2,152.93 (1,904.56, 2,382.73) |  | 9.50 | -0.28 (-0.30, -0.26) |  | 0.708 |
| Lesotho | 68.76 (63.41, 73.71) | 2,492.82 (2,311.50, 2,684.51) |  | 58.75 (53.72, 63.50) | 2,468.40 (2,284.21, 2,659.28) |  | -14.56 | -0.02 (-0.11, 0.06) |  | 0.507 |
| Liberia | 83.33 (77.23, 89.13) | 2,331.72 (2,136.35, 2,516.66) |  | 166.09 (154.81, 177.21) | 2,436.67 (2,251.29, 2,618.75) |  | 99.31 | 0.15 (0.13, 0.17) |  | 0.370 |
| Libya | 150.39 (137.55, 164.24) | 2,435.70 (2,256.80, 2,625.84) |  | 126.48 (117.98, 135.03) | 2,440.79 (2,248.95, 2,639.78) |  | -15.90 | -0.02 (-0.04, 0.00) |  | 0.709 |
| Lithuania | 60.80 (54.53, 66.68) | 2,483.46 (2,297.30, 2,669.26) |  | 32.80 (29.47, 35.99) | 2,369.25 (2,159.59, 2,568.94) |  | -46.05 | -0.22 (-0.29, -0.15) |  | 0.843 |
| Luxembourg | 3.33 (3.02, 3.65) | 2,460.41 (2,264.89, 2,641.09) |  | 5.03 (4.60, 5.52) | 2,471.32 (2,277.64, 2,688.36) |  | 51.15 | 0.02 (0.02, 0.03) |  | 0.895 |
| Macedonia | 39.51 (35.30, 43.49) | 2,574.05 (2,378.67, 2,762.01) |  | 27.56 (24.92, 30.28) | 2,541.52 (2,350.94, 2,738.27) |  | -30.25 | -0.05 (-0.05, -0.05) |  | 0.744 |
| Madagascar | 543.34 (507.50, 577.42) | 2,601.52 (2,402.22, 2,808.91) |  | 1,024.09 (948.58, 1,096.85) | 2,638.41 (2,435.63, 2,838.39) |  | 88.48 | 0.01 (-0.01, 0.02) |  | 0.396 |
| Malawi | 446.35 (417.81, 474.92) | 2,497.97 (2,293.48, 2,687.40) |  | 696.42 (641.78, 740.06) | 2,415.06 (2,202.24, 2,610.09) |  | 56.02 | -0.11 (-0.12, -0.11) |  | 0.384 |
| Malaysia | 454.26 (405.22, 502.68) | 2,499.59 (2,309.20, 2,682.24) |  | 536.68 (482.26, 590.80) | 2,474.53 (2,300.73, 2,652.07) |  | 18.14 | 0.02 (-0.16, 0.19) |  | 0.737 |
| Maldives | 7.27 (6.46, 8.17) | 2,501.17 (2,275.50, 2,762.86) |  | 8.64 (7.75, 9.43) | 2,534.42 (2,345.61, 2,720.74) |  | 18.74 | 0.02 (-0.01, 0.05) |  | 0.562 |
| Mali | 409.96 (379.42, 440.42) | 2,542.43 (2,352.24, 2,748.81) |  | 967.25 (895.32, 1,039.96) | 2,524.66 (2,330.35, 2,725.83) |  | 135.94 | -0.02 (-0.03, -0.01) |  | 0.263 |
| Malta | 4.24 (3.76, 4.71) | 2,540.21 (2,359.30, 2,736.54) |  | 3.98 (3.59, 4.36) | 2,473.45 (2,279.68, 2,660.54) |  | -6.19 | 0.04 (-0.02, 0.11) |  | 0.801 |
| Marshall Islands | 1.78 (1.63, 1.93) | 2,176.59 (2,005.06, 2,353.40) |  | 1.54 (1.42, 1.67) | 2,006.80 (1,808.56, 2,198.79) |  | -13.24 | -0.63 (-0.76, -0.51) |  | 0.544 |
| Mauritania | 90.96 (84.51, 97.54) | 2,208.34 (2,039.47, 2,392.19) |  | 138.46 (129.64, 147.22) | 1,954.19 (1,741.62, 2,150.80) |  | 52.22 | -0.46 (-0.48, -0.45) |  | 0.496 |
| Mauritius | 23.97 (21.82, 26.25) | 2,356.48 (2,167.49, 2,542.73) |  | 16.14 (14.70, 17.66) | 2,358.15 (2,173.08, 2,540.48) |  | -32.68 | -0.02 (-0.05, 0.00) |  | 0.705 |
| Mexico | 2,900.12 (2,679.80, 3,109.16) | 2,277.66 (2,087.73, 2,456.76) |  | 2,801.24 (2,614.23, 2,986.46) | 2,294.72 (2,096.55, 2,477.93) |  | -3.41 | 0.02 (0.02, 0.03) |  | 0.649 |
| Moldova | 93.83 (84.74, 102.73) | 2,444.71 (2,259.71, 2,631.80) |  | 49.15 (44.79, 53.20) | 2,295.20 (2,093.87, 2,494.47) |  | -47.61 | -0.27 (-0.31, -0.24) |  | 0.696 |
| Monaco | 0.23 (0.21, 0.25) | 2,216.81 (2,041.80, 2,397.47) |  | 0.27 (0.25, 0.30) | 2,151.04 (1,970.54, 2,331.61) |  | 21.04 | -0.46 (-0.67, -0.24) |  | 0.902 |
| Mongolia | 79.83 (73.45, 86.32) | 2,400.46 (2,209.59, 2,593.76) |  | 90.70 (82.43, 98.31) | 2,401.86 (2,220.98, 2,582.35) |  | 13.61 | 0.01 (-0.01, 0.04) |  | 0.606 |
| Montenegro | 11.18 (9.90, 12.39) | 2,369.12 (2,187.90, 2,559.30) |  | 8.21 (7.35, 9.00) | 2,361.79 (2,172.35, 2,552.46) |  | -26.61 | -0.06 (-0.12, -0.01) |  | 0.791 |
| Morocco | 823.10 (757.59, 901.14) | 2,278.39 (2,087.31, 2,464.13) |  | 786.96 (730.07, 842.88) | 2,270.64 (2,066.38, 2,462.39) |  | -4.39 | -0.02 (-0.03, -0.01) |  | 0.548 |
| Mozambique | 584.10 (542.61, 624.39) | 2,320.79 (2,143.29, 2,505.01) |  | 1,298.03 (1,211.69, 1,387.90) | 2,238.24 (2,037.50, 2,429.61) |  | 122.23 | -0.07 (-0.12, -0.02) |  | 0.307 |
| Myanmar | 1,058.84 (942.18, 1,178.91) | 2,333.57 (2,144.11, 2,520.76) |  | 1,008.74 (903.09, 1,108.88) | 2,322.16 (2,134.40, 2,525.99) |  | -4.73 | -0.05 (-0.07, -0.04) |  | 0.521 |
| Namibia | 55.45 (51.44, 59.28) | 2,397.14 (2,210.86, 2,612.41) |  | 76.98 (71.78, 82.35) | 2,365.76 (2,180.00, 2,552.80) |  | 38.84 | -0.06 (-0.08, -0.04) |  | 0.612 |
| Nauru | 0.29 (0.26, 0.33) | 2,383.51 (2,195.58, 2,567.20) |  | 0.27 (0.24, 0.30) | 2,514.64 (2,318.49, 2,683.78) |  | -8.75 | 0.18 (0.17, 0.19) |  | 0.618 |
| Nepal | 725.57 (670.36, 778.30) | 2,350.54 (2,167.85, 2,525.70) |  | 770.96 (716.91, 824.90) | 2,298.54 (2,110.48, 2,489.83) |  | 6.26 | -0.08 (-0.09, -0.08) |  | 0.422 |
| Netherlands | 127.36 (114.86, 140.75) | 2,282.62 (2,092.67, 2,471.65) |  | 133.93 (121.95, 146.86) | 2,282.00 (2,079.76, 2,474.88) |  | 5.16 | 0.00 (-0.01, 0.00) |  | 0.883 |
| New Zealand | 30.53 (27.09, 34.16) | 2,245.20 (2,055.09, 2,413.92) |  | 40.05 (35.87, 44.33) | 2,034.23 (1,819.99, 2,213.63) |  | 31.19 | -0.32 (-0.35, -0.28) |  | 0.840 |
| Nicaragua | 161.11 (148.92, 175.14) | 2,418.00 (2,214.92, 2,609.57) |  | 174.83 (162.56, 186.95) | 2,416.28 (2,227.24, 2,603.20) |  | 8.51 | 0.02 (-0.01, 0.05) |  | 0.517 |
| Niger | 401.93 (371.63, 432.06) | 2,393.85 (2,212.76, 2,592.72) |  | 1,143.28 (1,057.54, 1,227.37) | 2,367.09 (2,180.29, 2,562.11) |  | 184.45 | -0.02 (-0.03, 0.00) |  | 0.162 |
| Nigeria | 3,589.77 (3,295.33, 3,875.24) | 2,237.58 (2,054.88, 2,426.23) |  | 7,883.23 (7,272.14, 8,486.99) | 2,078.43 (1,876.58, 2,275.57) |  | 119.60 | -0.40 (-0.63, -0.16) |  | 0.515 |
| Niue | 0.05 (0.05, 0.06) | 2,238.91 (2,042.81, 2,422.08) |  | 0.03 (0.03, 0.03) | 2,285.82 (2,089.20, 2,484.68) |  | -45.86 | 0.06 (0.01, 0.12) |  | 0.711 |
| North Korea | 492.48 (430.67, 548.49) | 2,368.87 (2,168.98, 2,562.08) |  | 380.26 (341.88, 419.18) | 2,408.55 (2,221.24, 2,589.27) |  | -22.79 | 0.03 (0.00, 0.07) |  | 0.558 |
| Northern Mariana Islands | 0.93 (0.84, 1.03) | 2,390.37 (2,200.13, 2,591.03) |  | 0.60 (0.54, 0.65) | 2,493.27 (2,311.12, 2,678.35) |  | -36.25 | 0.13 (0.12, 0.15) |  | 0.771 |
| Norway | 37.31 (33.62, 41.18) | 2,281.59 (2,088.09, 2,484.36) |  | 45.02 (40.71, 49.35) | 2,423.44 (2,261.01, 2,606.94) |  | 20.68 | 0.21 (0.04, 0.38) |  | 0.913 |
| Oman | 67.73 (62.13, 73.31) | 2,273.44 (2,095.75, 2,466.06) |  | 97.24 (89.79, 104.81) | 2,452.16 (2,270.89, 2,632.53) |  | 43.57 | 0.28 (0.27, 0.30) |  | 0.783 |
| Pakistan | 4,400.89 (4,089.42, 4,737.49) | 2,261.96 (2,092.97, 2,448.65) |  | 7,214.98 (6,658.68, 7,786.29) | 2,380.57 (2,207.92, 2,558.12) |  | 63.94 | 0.16 (0.13, 0.18) |  | 0.449 |
| Palau | 0.33 (0.29, 0.36) | 2,263.78 (2,089.55, 2,426.89) |  | 0.26 (0.23, 0.28) | 2,420.45 (2,247.89, 2,603.14) |  | -21.97 | 0.31 (0.27, 0.35) |  | 0.738 |
| Palestine | 86.63 (78.80, 94.50) | 2,257.25 (2,089.93, 2,447.22) |  | 157.54 (145.69, 167.74) | 2,389.13 (2,195.09, 2,594.72) |  | 81.86 | 0.20 (0.18, 0.22) |  | 0.588 |
| Panama | 72.99 (67.58, 78.91) | 2,592.58 (2,413.05, 2,772.65) |  | 101.14 (93.78, 108.83) | 2,535.00 (2,356.27, 2,708.27) |  | 38.57 | -0.06 (-0.07, -0.05) |  | 0.686 |
| Papua New Guinea | 124.07 (111.43, 137.01) | 2,587.37 (2,415.11, 2,753.38) |  | 308.44 (276.30, 338.97) | 2,578.67 (2,389.98, 2,757.56) |  | 148.59 | -0.01 (-0.01, -0.01) |  | 0.394 |
| Paraguay | 122.33 (111.37, 133.96) | 2,581.62 (2,405.34, 2,766.57) |  | 137.25 (122.98, 150.14) | 2,528.54 (2,338.38, 2,718.61) |  | 12.19 | -0.06 (-0.07, -0.05) |  | 0.638 |
| Peru | 709.16 (658.35, 764.50) | 2,586.09 (2,412.60, 2,750.30) |  | 795.50 (735.89, 856.76) | 2,545.89 (2,365.11, 2,721.24) |  | 12.17 | -0.04 (-0.05, -0.03) |  | 0.648 |
| Philippines | 1,624.19 (1,458.34, 1,801.55) | 2,592.36 (2,412.51, 2,772.10) |  | 2,334.47 (2,097.55, 2,568.76) | 2,487.56 (2,281.33, 2,689.63) |  | 43.73 | -0.14 (-0.16, -0.12) |  | 0.623 |
| Poland | 627.63 (562.85, 689.00) | 2,570.60 (2,386.78, 2,759.73) |  | 424.59 (383.07, 464.72) | 2,479.88 (2,286.50, 2,672.35) |  | -32.35 | -0.11 (-0.13, -0.10) |  | 0.802 |
| Portugal | 124.75 (111.32, 139.11) | 2,584.26 (2,373.31, 2,798.17) |  | 94.45 (85.30, 103.90) | 2,675.43 (2,480.57, 2,864.63) |  | -24.29 | 0.14 (0.12, 0.15) |  | 0.743 |
| Puerto Rico | 78.19 (71.49, 84.56) | 2,575.70 (2,371.77, 2,782.97) |  | 46.05 (42.74, 49.14) | 2,686.34 (2,478.55, 2,866.64) |  | -41.10 | 0.16 (0.15, 0.18) |  | 0.814 |
| Qatar | 10.89 (9.92, 11.84) | 2,580.52 (2,395.16, 2,782.12) |  | 46.14 (42.89, 49.14) | 2,685.14 (2,501.86, 2,862.41) |  | 323.67 | 0.15 (0.14, 0.17) |  | 0.830 |
| Romania | 390.37 (351.45, 427.03) | 2,573.13 (2,386.93, 2,775.61) |  | 206.86 (185.97, 227.01) | 2,680.88 (2,482.04, 2,865.09) |  | -47.01 | 0.15 (0.14, 0.16) |  | 0.760 |
| Russia | 2,551.97 (2,317.77, 2,772.52) | 2,434.15 (2,256.49, 2,623.54) |  | 2,130.75 (1,937.50, 2,302.86) | 2,535.29 (2,323.32, 2,728.06) |  | -16.51 | -0.10 (-0.20, 0.00) |  | 0.805 |
| Rwanda | 333.36 (310.52, 354.26) | 2,455.08 (2,269.35, 2,644.09) |  | 442.56 (409.53, 472.72) | 2,537.46 (2,337.20, 2,723.63) |  | 32.76 | 0.13 (0.11, 0.14) |  | 0.429 |
| Saint Kitts and Nevis | 1.10 (1.01, 1.19) | 2,562.80 (2,374.02, 2,750.29) |  | 1.02 (0.94, 1.10) | 2,675.19 (2,464.00, 2,869.86) |  | -7.34 | 0.19 (0.12, 0.27) |  | 0.746 |
| Saint Lucia | 4.20 (3.85, 4.56) | 2,545.37 (2,365.67, 2,734.67) |  | 2.85 (2.65, 3.05) | 2,683.06 (2,466.15, 2,854.02) |  | -31.99 | 0.26 (0.20, 0.32) |  | 0.670 |
| Saint Vincent and the Grenadines | 3.30 (3.05, 3.57) | 2,072.55 (1,877.61, 2,282.46) |  | 2.12 (1.94, 2.28) | 1,791.16 (1,595.36, 2,006.34) |  | -35.79 | -0.42 (-0.48, -0.36) |  | 0.627 |
| Samoa | 3.96 (3.55, 4.37) | 2,566.32 (2,357.01, 2,774.89) |  | 4.59 (4.11, 5.05) | 2,681.86 (2,478.81, 2,874.64) |  | 15.81 | 0.16 (0.15, 0.17) |  | 0.641 |
| San Marino | 0.21 (0.19, 0.23) | 2,574.31 (2,371.03, 2,772.19) |  | 0.26 (0.24, 0.29) | 2,682.61 (2,478.50, 2,870.65) |  | 26.29 | 0.17 (0.16, 0.19) |  | 0.884 |
| Sao Tome and Principe | 5.15 (4.79, 5.50) | 1,857.92 (1,647.38, 2,064.43) |  | 6.31 (5.92, 6.70) | 1,800.64 (1,602.08, 2,004.15) |  | 22.59 | -0.10 (-0.11, -0.09) |  | 0.502 |
| Saudi Arabia | 515.81 (469.86, 558.41) | 2,494.51 (2,333.11, 2,690.81) |  | 619.50 (571.21, 660.87) | 2,676.75 (2,472.55, 2,867.45) |  | 20.10 | 0.20 (0.19, 0.21) |  | 0.805 |
| Senegal | 355.48 (330.62, 380.59) | 2,562.88 (2,377.28, 2,759.96) |  | 549.87 (510.19, 587.26) | 2,683.48 (2,481.72, 2,870.38) |  | 54.68 | 0.18 (0.17, 0.19) |  | 0.389 |
| Serbia | 171.90 (155.23, 187.60) | 2,570.61 (2,373.43, 2,762.11) |  | 127.26 (115.28, 137.22) | 2,681.16 (2,484.08, 2,863.93) |  | -25.97 | 0.16 (0.15, 0.18) |  | 0.767 |
| Seychelles | 1.54 (1.37, 1.71) | 2,571.04 (2,371.14, 2,762.68) |  | 1.53 (1.37, 1.68) | 2,683.13 (2,482.61, 2,854.59) |  | -0.52 | 0.17 (0.16, 0.18) |  | 0.724 |
| Sierra Leone | 156.05 (145.02, 167.31) | 2,595.62 (2,387.79, 2,790.10) |  | 308.31 (287.26, 329.84) | 2,610.46 (2,399.40, 2,796.12) |  | 97.58 | 0.02 (0.02, 0.03) |  | 0.347 |
| Singapore | 32.32 (29.04, 35.69) | 2,581.02 (2,359.18, 2,778.58) |  | 53.41 (48.36, 58.39) | 2,604.38 (2,393.36, 2,803.60) |  | 65.23 | 0.02 (0.01, 0.03) |  | 0.861 |
| Slovakia | 86.78 (77.66, 96.42) | 2,585.66 (2,373.16, 2,785.79) |  | 65.36 (58.80, 71.76) | 2,613.98 (2,432.21, 2,802.66) |  | -24.69 | 0.04 (0.03, 0.04) |  | 0.812 |
| Slovenia | 28.69 (25.66, 31.56) | 2,479.22 (2,293.17, 2,667.51) |  | 23.02 (20.67, 25.27) | 2,481.95 (2,290.40, 2,665.48) |  | -19.76 | 0.00 (-0.01, 0.01) |  | 0.840 |
| Solomon Islands | 10.93 (9.68, 12.25) | 2,590.90 (2,387.49, 2,792.69) |  | 18.55 (16.63, 20.49) | 2,611.91 (2,428.95, 2,799.98) |  | 69.65 | 0.02 (0.02, 0.03) |  | 0.407 |
| Somalia | 319.41 (300.82, 339.45) | 2,592.75 (2,380.14, 2,791.73) |  | 936.42 (866.47, 999.75) | 2,615.03 (2,413.83, 2,805.83) |  | 193.17 | 0.01 (0.00, 0.02) |  | 0.081 |
| South Africa | 1,139.73 (1,054.53, 1,224.55) | 2,610.81 (2,420.01, 2,793.29) |  | 1,296.51 (1,189.32, 1,398.70) | 2,609.12 (2,414.15, 2,784.41) |  | 13.76 | 0.01 (0.01, 0.01) |  | 0.678 |
| South Korea | 745.33 (679.76, 818.62) | 2,612.05 (2,420.63, 2,797.34) |  | 565.86 (511.56, 622.56) | 2,615.87 (2,432.91, 2,794.55) |  | -24.08 | 0.01 (0.01, 0.01) |  | 0.878 |
| South Sudan | 253.75 (237.03, 270.52) | 2,585.73 (2,406.85, 2,750.93) |  | 392.80 (365.39, 418.97) | 2,604.22 (2,420.84, 2,774.67) |  | 54.80 | 0.02 (0.01, 0.03) |  | 0.363 |
| Spain | 370.55 (350.47, 389.68) | 2,614.88 (2,418.22, 2,799.91) |  | 398.50 (363.92, 434.65) | 2,574.23 (2,392.51, 2,748.70) |  | 7.54 | -0.05 (-0.05, -0.04) |  | 0.767 |
| Sri Lanka | 310.12 (274.63, 344.94) | 2,612.00 (2,420.88, 2,797.53) |  | 279.23 (250.52, 310.56) | 2,621.97 (2,434.35, 2,792.16) |  | -9.96 | 0.01 (0.00, 0.01) |  | 0.690 |
| Sudan | 808.43 (739.40, 884.99) | 2,606.65 (2,403.65, 2,796.52) |  | 1,336.79 (1,235.09, 1,435.34) | 2,607.35 (2,426.25, 2,790.13) |  | 65.36 | 0.01 (0.01, 0.01) |  | 0.515 |
| Suriname | 10.79 (9.94, 11.64) | 2,610.15 (2,414.63, 2,794.17) |  | 11.86 (10.80, 12.78) | 2,609.63 (2,441.23, 2,788.36) |  | 9.86 | -0.01 (-0.01, 0.00) |  | 0.636 |
| Sweden | 54.92 (49.35, 60.55) | 2,608.93 (2,417.30, 2,805.52) |  | 59.83 (54.42, 65.35) | 2,610.67 (2,439.81, 2,779.43) |  | 8.95 | 0.00 (-0.01, 0.00) |  | 0.872 |
| Switzerland | 48.06 (43.45, 52.26) | 2,612.49 (2,417.29, 2,791.45) |  | 61.17 (55.86, 66.26) | 2,613.40 (2,426.98, 2,788.14) |  | 27.27 | 0.00 (0.00, 0.01) |  | 0.929 |
| Syrian Arab Republic | 518.53 (472.98, 557.79) | 2,606.45 (2,408.79, 2,794.76) |  | 323.26 (301.20, 345.39) | 2,605.71 (2,433.00, 2,779.37) |  | -37.66 | 0.00 (0.00, 0.00) |  | 0.619 |
| Taiwan (Province of China) | 360.26 (322.41, 393.00) | 2,609.84 (2,412.82, 2,800.31) |  | 257.81 (236.29, 279.87) | 2,627.76 (2,445.02, 2,805.88) |  | -28.44 | 0.01 (0.00, 0.02) |  | 0.868 |
| Tajikistan | 213.70 (195.13, 232.69) | 2,611.88 (2,421.00, 2,797.44) |  | 297.21 (274.77, 321.00) | 2,606.54 (2,423.49, 2,778.79) |  | 39.08 | 0.00 (0.00, 0.01) |  | 0.539 |
| Thailand | 1,206.32 (1,124.80, 1,285.50) | 2,608.34 (2,412.93, 2,786.12) |  | 2,363.82 (2,200.12, 2,521.04) | 2,601.56 (2,428.42, 2,769.91) |  | 1.92 | -0.01 (-0.01, 0.00) |  | 0.423 |
| Timor-Leste | 774.93 (706.86, 845.23) | 2,611.95 (2,421.11, 2,797.46) |  | 789.85 (718.58, 855.83) | 2,617.08 (2,436.40, 2,796.56) |  | 32.78 | 0.01 (0.00, 0.01) |  | 0.687 |
| Togo | 25.18 (22.32, 28.38) | 2,472.75 (2,290.39, 2,643.13) |  | 33.43 (29.53, 37.16) | 2,476.64 (2,298.05, 2,654.70) |  | 67.07 | -0.01 (-0.03, 0.01) |  | 0.514 |
| Tokelau | 168.55 (156.12, 180.10) | 2,611.70 (2,421.44, 2,797.91) |  | 281.59 (261.76, 299.51) | 2,587.85 (2,417.88, 2,756.97) |  | -32.03 | -0.03 (-0.03, -0.02) |  | 0.417 |
| Tonga | 0.05 (0.04, 0.05) | 2,601.88 (2,410.17, 2,789.54) |  | 0.03 (0.03, 0.04) | 2,618.46 (2,428.10, 2,797.81) |  | -7.26 | 0.03 (0.02, 0.03) |  | 0.626 |
| Trinidad and Tobago | 2.57 (2.29, 2.85) | 2,612.05 (2,420.90, 2,797.87) |  | 2.38 (2.12, 2.64) | 2,613.09 (2,443.57, 2,792.03) |  | -28.04 | 0.00 (0.00, 0.00) |  | 0.636 |
| Tunisia | 31.31 (28.68, 33.74) | 2,603.72 (2,397.15, 2,792.57) |  | 22.53 (20.41, 24.43) | 2,606.71 (2,419.86, 2,772.27) |  | -14.99 | 0.00 (-0.01, 0.00) |  | 0.757 |
| Turkey | 264.88 (244.55, 287.03) | 1,955.12 (1,759.72, 2,164.69) |  | 225.19 (208.08, 242.56) | 1,979.09 (1,766.71, 2,170.50) |  | -20.41 | 0.03 (-0.02, 0.08) |  | 0.672 |
| Turkmenistan | 1,658.59 (1,521.66, 1,804.51) | 2,262.28 (2,054.26, 2,462.75) |  | 1,320.05 (1,217.23, 1,417.85) | 2,175.44 (1,944.50, 2,377.62) |  | -2.77 | -0.14 (-0.15, -0.13) |  | 0.748 |
| Tuvalu | 135.35 (122.92, 146.99) | 1,905.14 (1,696.75, 2,118.52) |  | 131.61 (120.07, 142.86) | 1,962.36 (1,764.07, 2,158.96) |  | 4.12 | 0.08 (0.04, 0.12) |  | 0.670 |
| Uganda | 0.23 (0.21, 0.26) | 1,520.27 (1,326.99, 1,711.23) |  | 0.24 (0.22, 0.27) | 1,488.96 (1,289.55, 1,694.07) |  | 105.01 | -0.04 (-0.07, -0.02) |  | 0.589 |
| Ukraine | 884.80 (823.84, 940.08) | 1,892.39 (1,690.03, 2,088.60) |  | 1,813.92 (1,695.79, 1,931.73) | 1,949.31 (1,726.78, 2,147.48) |  | -32.98 | 0.08 (0.03, 0.14) |  | 0.404 |
| United Arab Emirates | 793.78 (711.82, 863.70) | 997.92 (877.80, 1,118.39) |  | 532.03 (481.90, 573.68) | 966.91 (860.31, 1,085.44) |  | 145.54 | -0.09 (-0.10, -0.08) |  | 0.736 |
| United Kingdom | 48.94 (44.43, 53.20) | 1,970.19 (1,773.70, 2,154.88) |  | 120.16 (110.33, 128.50) | 2,013.76 (1,799.55, 2,223.73) |  | 6.54 | 0.06 (0.04, 0.09) |  | 0.880 |
| Tanzania | 553.65 (499.73, 607.28) | 1,940.80 (1,745.92, 2,148.78) |  | 589.88 (535.37, 645.43) | 1,977.12 (1,763.54, 2,186.44) |  | 95.95 | 0.04 (-0.01, 0.08) |  | 0.847 |
| United States of America | 2,658.31 (2,372.31, 2,936.03) | 1,874.98 (1,680.18, 2,065.98) |  | 3,212.06 (2,884.26, 3,526.52) | 1,957.54 (1,748.90, 2,161.38) |  | 20.83 | 0.15 (0.10, 0.19) |  | 0.859 |
| United States Virgin Islands | 2.51 (2.28, 2.73) | 1,923.07 (1,719.91, 2,122.55) |  | 1.62 (1.45, 1.76) | 1,979.80 (1,763.87, 2,178.00) |  | -35.72 | 0.08 (0.04, 0.13) |  | 0.799 |
| Uruguay | 67.37 (60.22, 73.35) | 2,250.11 (2,044.81, 2,446.78) |  | 56.24 (50.37, 61.45) | 2,178.89 (1,949.75, 2,388.99) |  | -16.52 | -0.11 (-0.12, -0.10) |  | 0.697 |
| Uzbekistan | 800.92 (742.47, 861.20) | 2,338.41 (2,141.88, 2,535.62) |  | 937.40 (868.36, 1,005.77) | 2,217.84 (1,999.59, 2,428.08) |  | 17.04 | -0.19 (-0.20, -0.17) |  | 0.631 |
| Vanuatu | 4.66 (4.14, 5.21) | 1,002.56 (884.52, 1,130.84) |  | 7.99 (7.15, 8.79) | 968.02 (858.87, 1,098.29) |  | 71.35 | -0.09 (-0.10, -0.09) |  | 0.485 |
| Venezuela | 625.76 (580.05, 675.87) | 2,004.54 (1,801.39, 2,203.78) |  | 616.76 (572.19, 658.97) | 2,008.65 (1,796.21, 2,206.54) |  | -1.44 | -0.03 (-0.07, 0.02) |  | 0.607 |
| Viet Nam | 2,109.55 (1,916.91, 2,302.64) | 2,032.24 (1,834.57, 2,250.54) |  | 1,681.33 (1,516.23, 1,845.36) | 2,042.22 (1,824.65, 2,255.30) |  | -20.30 | 0.01 (-0.03, 0.06) |  | 0.617 |
| Yemen | 595.68 (540.37, 651.86) | 2,289.84 (2,082.66, 2,484.32) |  | 1,035.24 (951.03, 1,114.34) | 2,181.61 (1,923.26, 2,409.66) |  | 73.79 | -0.17 (-0.19, -0.16) |  | 0.412 |
| Zambia | 376.17 (350.45, 401.77) | 2,573.42 (2,387.75, 2,776.56) |  | 729.02 (678.13, 774.46) | 2,670.61 (2,465.16, 2,857.70) |  | 93.80 | 0.15 (0.14, 0.16) |  | 0.505 |
| Zimbabwe | 440.85 (408.52, 472.02) | 2,404.66 (2,215.34, 2,605.78) |  | 541.84 (502.61, 579.45) | 2,478.51 (2,293.83, 2,655.16) |  | 22.91 | 0.10 (0.08, 0.11) |  | 0.476 |

ASIR: age-standardized rate; CI: confidence interval; EAPC: estimated annual percentage change; GBD: Global Burden of Disease; SDI: socio-demographic index; UI: uncertainty interval.
